# Supplementary material for: UBAP2 plays a role in bone homeostasis through the regulation of osteoblastogenesis and osteoclastogenesis
Source: Nat Commun. 2023 Jun 20;14:3668. doi: 10.1038/s41467-023-39448-8 (PMC10281941; doi:10.1038/s41467-023-39448-8)
Supplement: Supplementary file 1 — Supplementary Information [file 41467_2023_39448_MOESM1_ESM.pdf]

SUPPLEMENTARY INFORMATION:

UBAP2 plays a role in bone homeostasis through the regulation of osteoblastogenesis and osteoclastogenesis

Jeonghyun Kim, Bo-Young Kim, Jeong-Soo Lee, Yun-Mi Jeong, Hyun-Ju Cho, Eunkuk Park, Dowan Kim, Sung-Soo Kim, Bom-Taeck Kim, Yong Jun Choi, Ye-Yeon Won, Hyun-Seok Jin, Yoon-Sok Chung & Seon-Yong Jeong

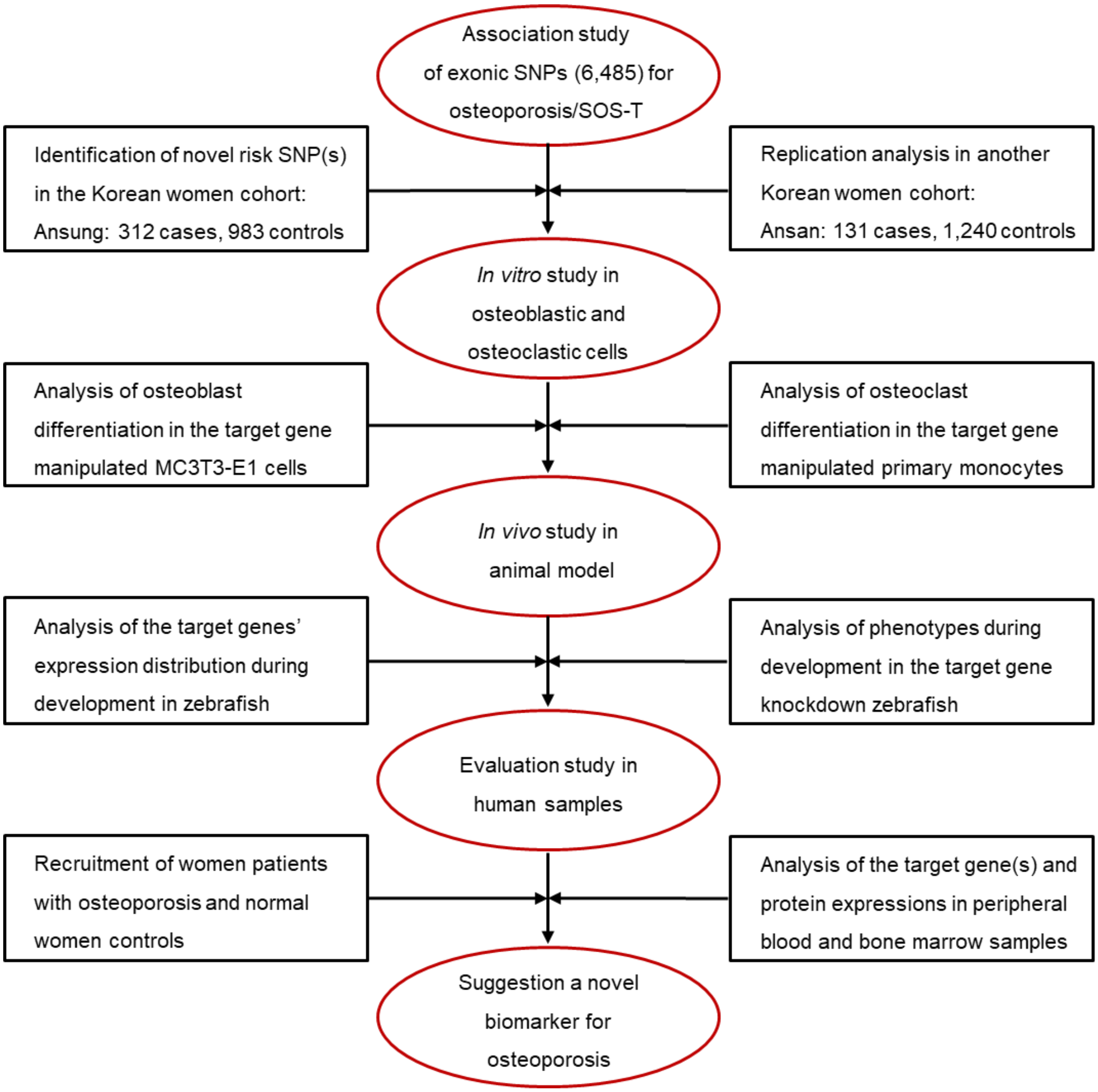

Supplementary Fig. 1. Study design. To identify novel biomarkers for the assessment of osteoporosis, a series of statistical and experimental analyses were carried out. First, an exome-wide association study was conducted using 6,485 whole-genome exonic single nucleotide polymorphisms (SNPs) associated with osteoporosis and speed of sound T-score (SOS T) at midshaft tibia and distal radius in 1,295 participants of a Korean women's cohort (Ansung)[1]. Replication analysis of the discovered significant SNPs was performed in a total of 1,371 subjects of another Korean women's cohort (Ansan). Next, *in vitro* functional analysis of the target gene corresponding to the identified SNP was performed by knockdown and overexpression of target the gene in osteoblastic MC3T3-E1 cells and osteoclastic-lineage primary-cultured monocytes. Next, expression distribution of the target gene during development was examined by mRNA *in situ* hybridization. Knockdown of the target gene was conducted in zebrafish embryos and then phenotype alterations in bone and cartilage during development were observed. Next, comparison analysis of mRNA levels of *UBAP2* and biomarkers involved in osteoblastogenesis and osteoclastogenesis was conducted using human bone marrow and peripheral blood samples from volunteers of women with osteoporosis and normal control women. In addition, protein levels of UBAP2 and osteocalcin were compared between blood plasmas of the two groups. Finally, by comprehensive analysis of all of the data, we suggested a novel biomarker available for clinical assessment of osteoporosis in women.

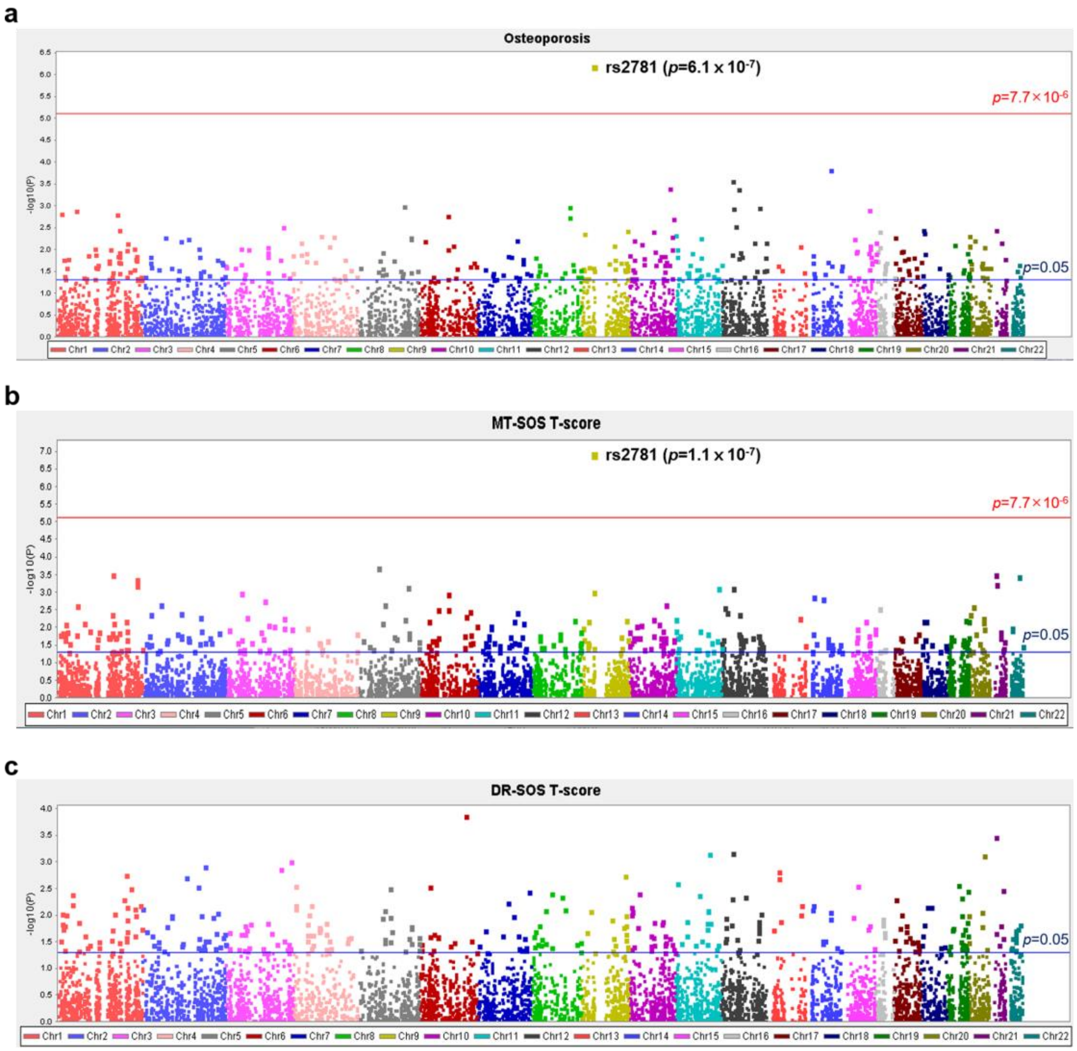

Supplementary Fig. 2. Manhattan plots of the exome-wide association studies of exonic SNPs associated with osteoporosis and bone density in the combined cohorts of Ansung and Ansan. a Results of case-control analysis of the exome-wide association study for osteoporosis. The statistical significance probability values (–log10 *p*-value) of the 6,485 exonic single nucleotide polymorphisms (SNPs) in each chromosome are plotted. The most significant rs2781 SNP is indicated with the *p*-value. **b, c** Results of quantitative trait analysis of the exome-wide association study for speed of sound (SOS) T-score at midshaft tibia (MT-SOS T score) and SOS T-score at distal radius (DR-SOS T-score). The most significant SNP (rs2781) is indicated with *p*-values. The blue line represents a minimum statistical significance *p*-value ($p < 0.05$), and the red line represents Bonferroni-corrected significance *p*-value ($p < 7.7\times10^{-6}$).

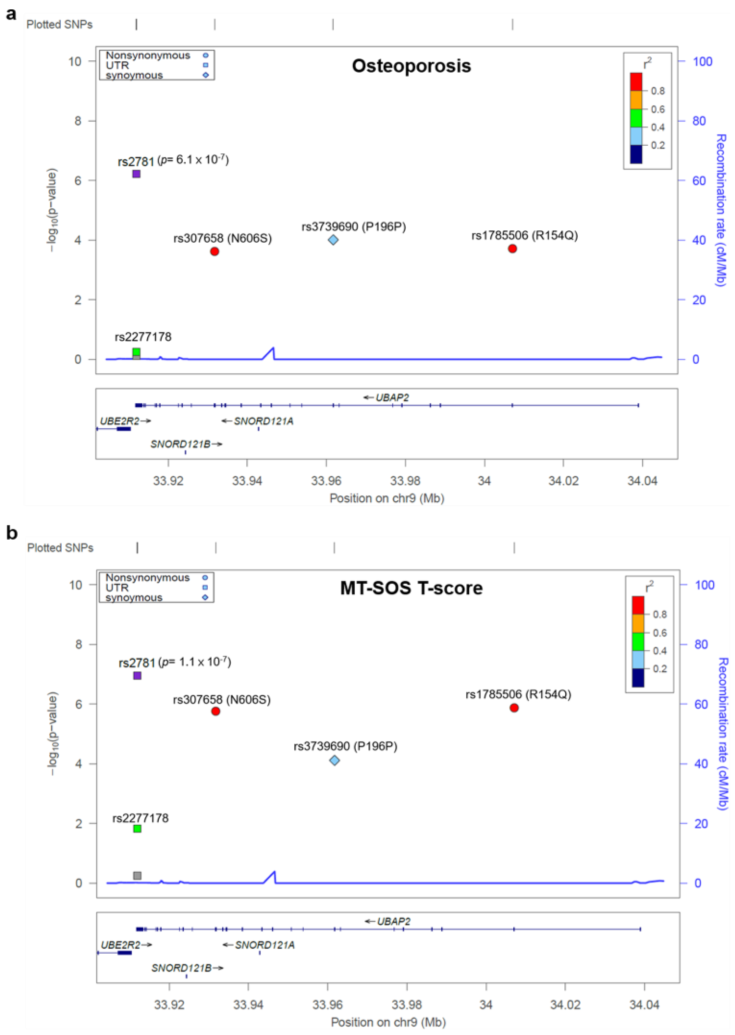

Supplementary Fig. 3. Signal plots of the exonic SNPs within *UBAP2* associated with osteoporosis and bone density in the combined cohorts of Ansung and Ansan. a Signal plot of rs2781 associated with osteoporosis and **b** the plot of rs2781 for speed of sound T-score at midshaft tibia (MT-SOS T score). The statistical significances (–log10 *p*-value) of the analyzed SNPs within *UBAP2* are plotted. rs2781 is the SNP most significantly associated with osteoporosis and speed of sound T-score at midshaft tibia (MT-SOS T score), and its correlated SNPs are shown in the indicated colors in accordance with levels of linkage disequilibrium (LD, r^2). Predicted functions of SNPs are indicated by different symbols. The recombination rate estimated from the HapMap CHB (Han Chinese from Beijing) and JPT (Japanese from Tokyo) population data is shown by a blue bar. The position (Mb) of each gene on human chromosome 9 (NCBI build 36) is shown at the bottom.

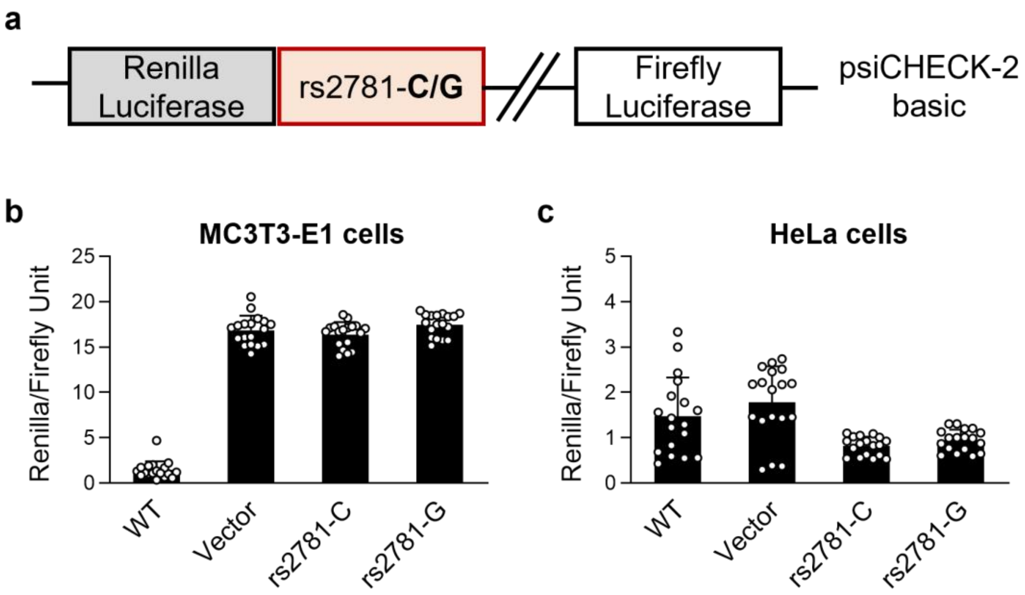

Supplementary Fig. 4. Report gene assay of the major and minor alleles of rs2781 SNP in human *UBAP2* in mouse pre-osteoblast MC3T3-E1 cells and human HeLa cells. a A schematic diagram of psiCHECK-2 basic luciferase reporter gene assay vector (Promega; Madison, WI, USA) and the position where the 3′ untranslated region (UTR) (811 bp) of human *UBAP2* (NM_001370066) was cloned. The construct containing the minor allele sequence (nucleotide G) of rs2781 SNP at 3′ UTR of *UBAP2* was constructed using the site-directed mutagenesis method with the QuickChange Lightning Site-Directed Mutagenesis Kit (Agilent Technologies; Santa Clara, CA, USA) according to the manufacturer's instructions. Two constructs were verified by DNA sequencing. **b, c** Luciferase reporter gene assay of rs2781 SNP in mouse pre-osteoblast MC3T3-E1 and human HeLa cells. Cells were transfected with psiCHECK-2 empty vector or psiCHECK-2 constructs containing the 3′ UTR of *UBAP2* for rs2781 C allele (rs2781-C) or rs2781 G allele (rs2781-G) constructs. Transfected cells were harvested and lysed by manually scraping with passive lysis buffer. The lysates were then mixed with LAR II followed by sequential injection of Stop & Glo Reagents of Dual-Luciferase Reporter Assay System (Promega), and apparent luminescence was measured using the luminometer (Turner Design Instrument; Sunnyvale, CA, USA). Luciferase activity was normalized to the Firefly luciferase activity. All experiments were repeated nine times with duplicated determinations. Significant differences among multiple groups were determined using a one-way ANOVA, followed by Tukey's honest significant difference post-hoc test. All data are presented as mean ± SD with dot plots. Source data are provided as a Source Data file.

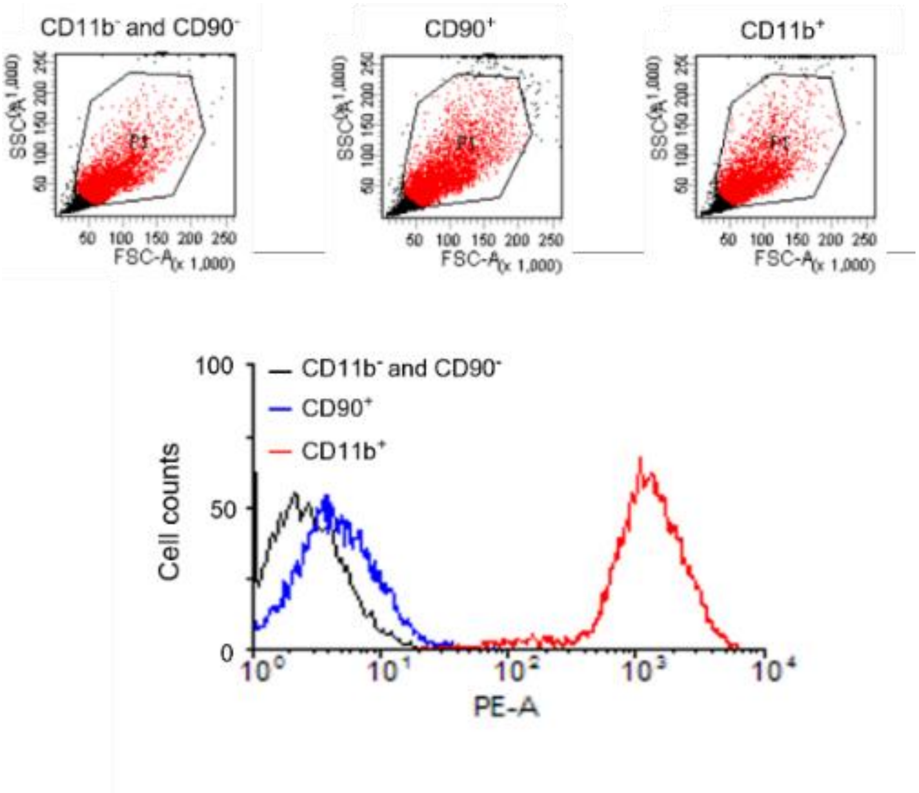

Supplementary Fig. 5. Confirmation of monocyte isolation from mouse bone marrow. Successfully isolated monocytes from mouse bone marrows were confirmed by immunophenotypic analysis with a monocyte-specific surface positive marker (PE-conjugated CD11b antibody, blue line) using fluorescence-activated cell sorting (FACS) analysis. The results of FACS gating plots and histogram of CD90 and CD11b were shown. The absence of contamination of mesenchymal stem cells (MSCs) was confirmed by an MSC-specific surface marker (PE-conjugated CD90 antibody, red line).

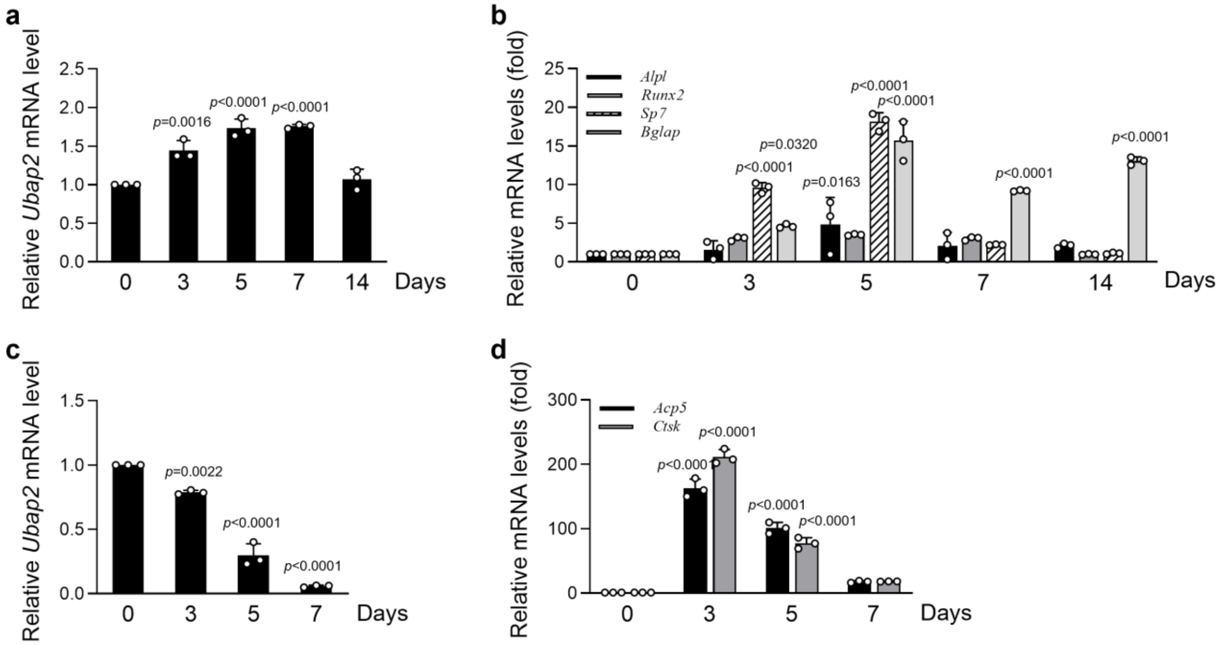

Supplementary Fig. 6. Analysis of mRNA expression patterns of *Ubap2* during osteoblastogenesis and osteoclastogenesis. a, b Quantification of mRNA levels of *Ubap2* and representative genes *Alpl*, *Runx2*, *Sp7*, and *Bglap*, involved in osteoblastogenesis in mouse pre-osteoblast MC3T3-E1 cells after induction of osteoblast differentiation. MC3T3-E1 cells were treated with 50 μg/mL ascorbic acid and 10 mM β-glycerophosphate for 14 days. Cells were harvested in the indicated days and total RNA was isolated. Quantitative reserve-transcription PCR (qRT-PCR) with gene specific primers was performed, and Relative mRNA level was determined by normalizing to mouse *Gusb* (*n* = 3 independent experiments; duplicate samples). Relative gene expression is presented as fold change relative to the non-induced control (Day 0). Significant differences among multiple groups were determined using a two-way ANOVA. Exact *p*-values representing comparison to Day 0 are shown. All data are shown as mean ± SD with dot-plots. **c, d** Quantification of mRNA levels of *Ubap2* and representative genes, *Acp5* and *Ctsk*, involved in osteoclastogenesis in primary-cultured mouse monocytes after induction of osteoclast differentiation. Monocytes were treated with 50 ng/mL RANKL and 30 ng/mL M-CSF for 7 days. Quantitative reserve-transcription PCR (qRT-PCR) with gene specific primers was performed, and Relative mRNA level was determined by normalizing to mouse *Gusb* (*n* = 3 independent experiments; duplicate samples). Relative gene expression is presented as fold change relative to the non-induced control (Day 0). Significant differences among multiple groups were determined using a two-way ANOVA. Exact *p*-values representing comparison to Day 0 are shown. All data are presented as mean ± SD with dot plots. Source data are provided as a Source Data file.

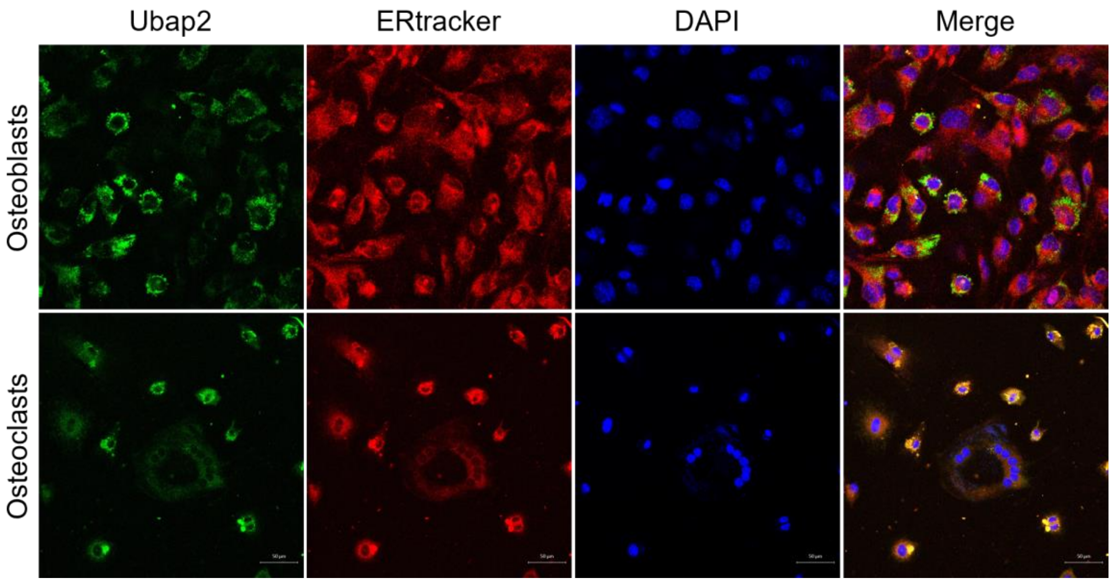

Supplementary Fig. 7. The localization of endogenous Ubap2 in osteoblasts and osteoclasts. Osteoblast differentiation-induced pre-osteoblast MC3T3-E1 cells and Osteoclast differentiation-induced primary-cultured mouse monocytes were incubated with ER tracker and DAPI for endoplasmic reticulum and nuclear staining, respectively, treated with anti-Ubap2 antibody and sequentially secondary antibody conjugated with FITC. Fluorescence was visualized with confocal microscopy using a Zeiss LSM 710 (Carl Zeiss Microscopy; Jena, Germany). All experiments were independently repeated twice with duplicated samples. Scale bar, 50 μm. Source data are provided as a Source Data file.

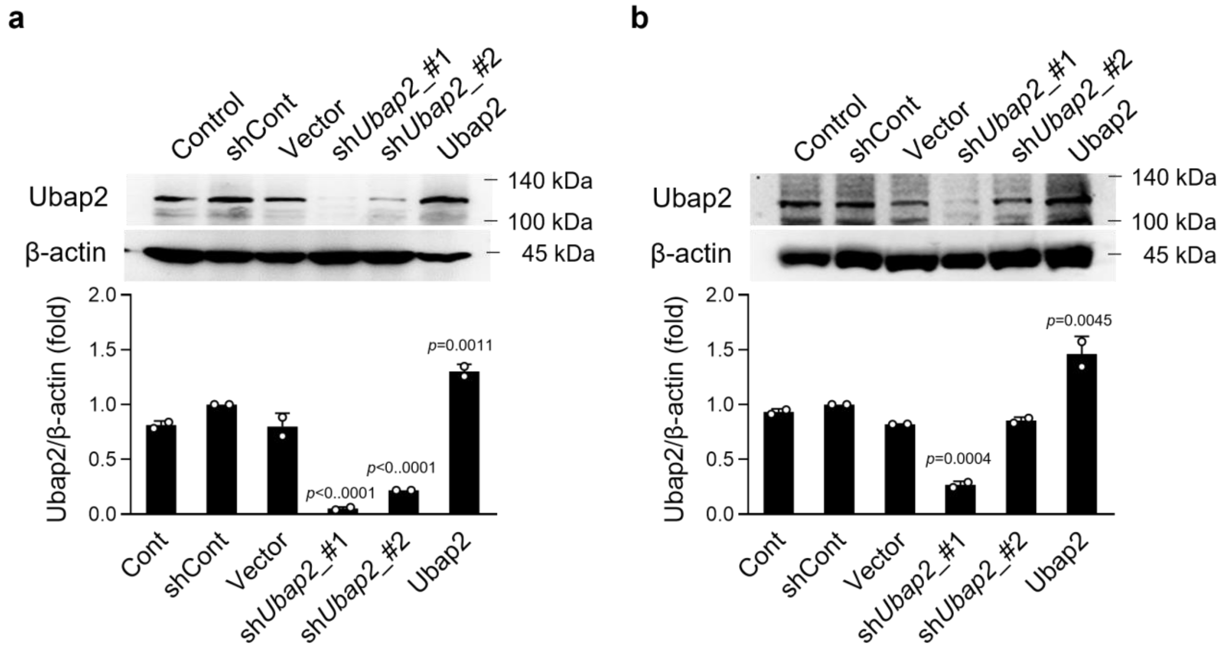

Supplementary Fig. 8. Schematic diagrams and evaluation of *Ubap2* knockdown and overexpression constructs. a, b Evaluation of *Ubap2* knockdown and overexpression constructs by western blotting of Ubap2 antibody. Pre-osteoblast MC3T3-E1 cells and primary monocytes were infected with the viral particles of pLKO.1-puro empty vector (shCont), pDON-5 Neo vector (Vector), *Ubap2* knockdown (sh*Ubap2*_#1 and sh*Ubap2*_#2) constructs, and *Ubap2* overexpression construct. The protein level intensities for quantitative analysis were normalized to β-actin using Image Processing and Analysis in Java (Image J) software (http://imagej.nih.gov/ij/). All experiments were independently repeated twice. Significant differences among multiple groups were determined using a one-way ANOVA, followed by Tukey's honest significant difference post-hoc test. Exact *p*-values representing comparison to short hairpin RNA control (shCon) are shown. All data are presented as mean ± SD with dot plots. Source data are provided as a Source Data file.

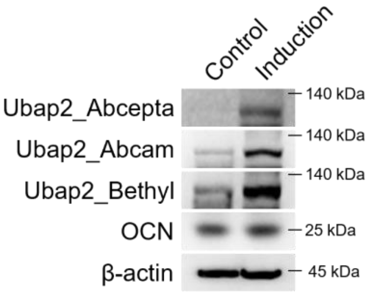

Supplementary Fig. 9. Specificity test for the Ubap2 antibodies in pre-osteoblast MC3T3-E1 cells. For osteoblast differentiation induction, cells were incubated with the osteoblastic medium containing ascorbic acid and β-glycerophosphate for 4 days. The three kinds of Ubap2 antibodies (Abcepta, Abcam, and Bethyl Laboratories, Inc.) that recognize different epitope regions of Ubap2 protein. Osteocalcin (OCN) and β-actin antibodies were used as controls. All experiments were independently repeated twice. Source data are provided as a Source Data file.

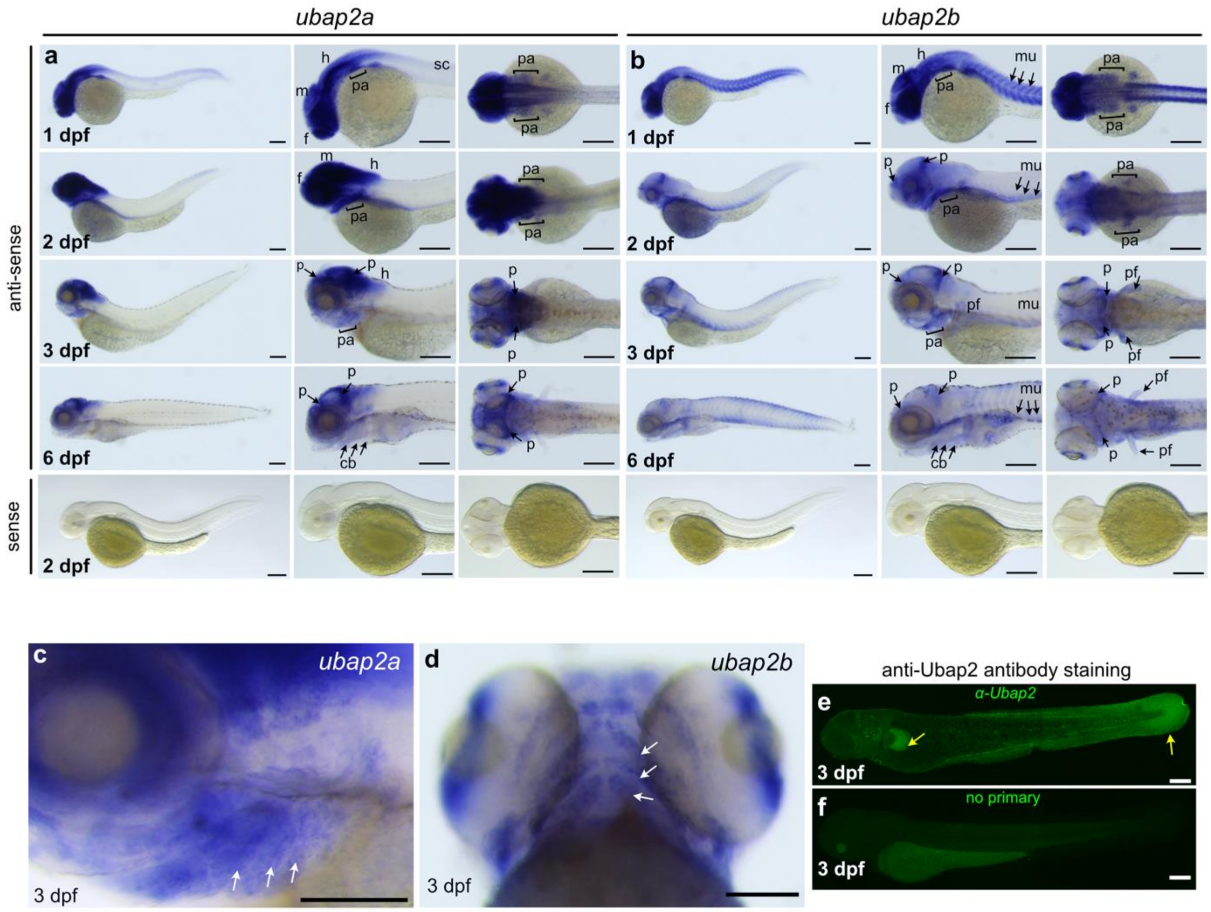

Supplementary Fig. 10. Expression of *ubap2a* and *ubap2b* in the developing zebrafish. a-d Representative images of the whole-mount *in situ* hybridization of two zebrafish homologues of human *UBAP2*, *ubap2a* and *ubap2b,* during development. **a** *ubap2a* is mainly expressed in the brain and pharyngeal arches where bone and cartilage develop (black arrows). **b** *ubap2b* is expressed in the brain, muscles, pectoral fins and proliferating zone of brain as well as pharyngeal arches (black arrows). Left panels show the whole body of developing zebrafish (1, 2, 3 and 6 dpf) with lateral view, and middle and right panels show enlarged images of the left panels with lateral view (middle) and dorsal view (right). **c, d** Expression of *ubap2a* mRNA (**c**, lateral view) and *ubap2b* mRNA (**d**, ventral view) in the pharyngeal arches (white arrows in **c, d**) of developing bone structures. **e, f** Whole-mount immunofluorescence of Ubap2 protein in the zebrafish 3 dpf larvae. **e** Confocal fluorescence images of immunostained zebrafish with Ubap2 antibody. Yellow arrows indicate strong expression of Ubap2 in the pectoral and caudal fins. **f** Control experiment without primary antibody confirming that the staining in **e** is not autofluorescence. cb, ceratobranchials; dpf, days post fertilization; f, forebrain; h, hindbrain; m, midbrain; mu, muscle; p, proliferating zone of the brain; pa, pharyngeal arches; pf, pectoral fins. Scale bars = 200 μm. Each experiment was independently repeated twice. Source data are provided as a Source Data file.

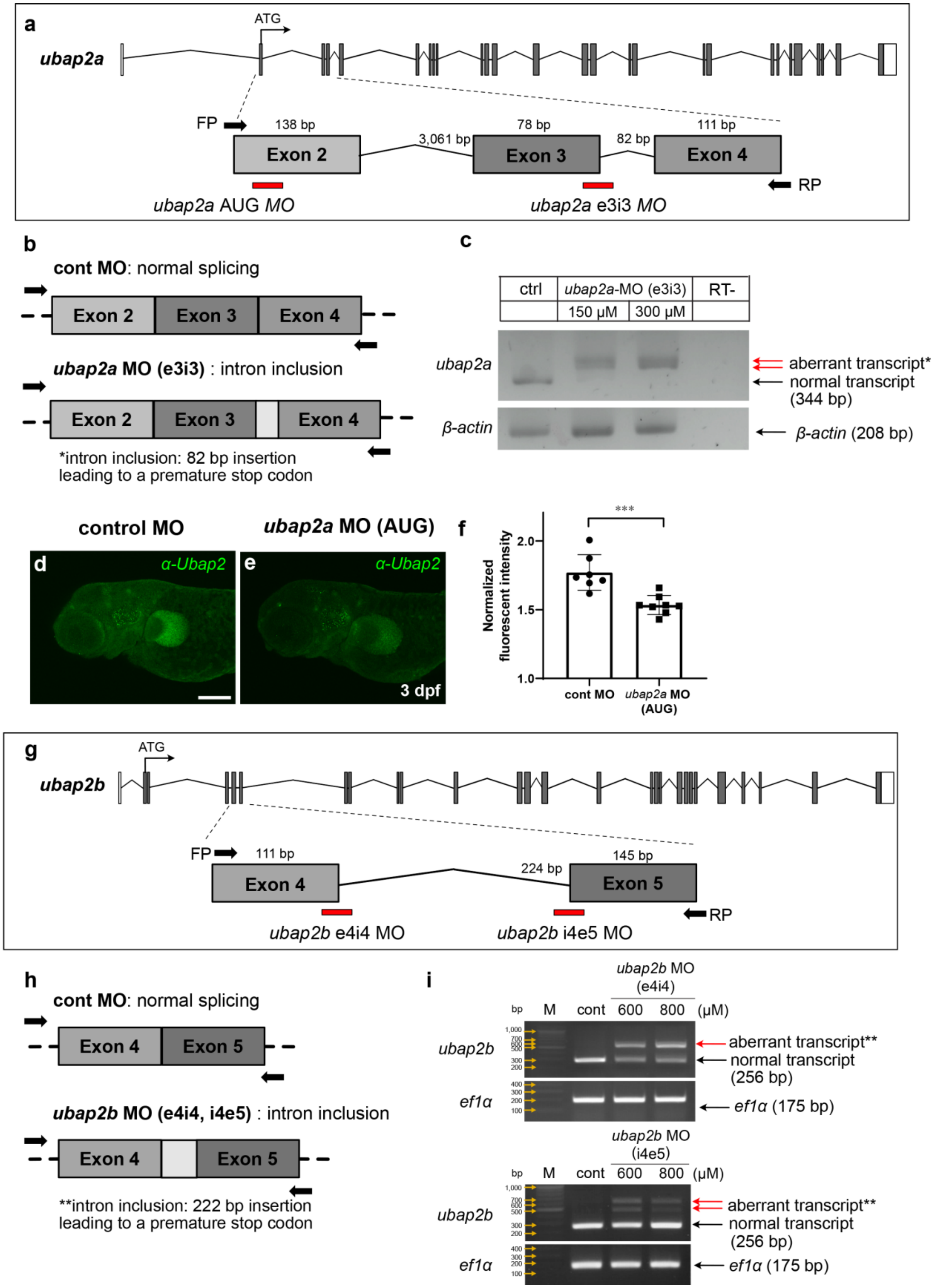
a
ubap2a
ATG
FP
138 bp
Exon 2
3,061 bp
78 bp
Exon 3
82 bp
111 bp
Exon 4
RP
ubap2a AUG MO
ubap2a e3i3 MO
b
cont MO: normal splicing
Exon 2
Exon 3
Exon 4
ubap2a MO (e3i3) : intron inclusion
Exon 2
Exon 3
Exon 4
*intron inclusion: 82 bp insertion
leading to a premature stop codon
c
ctrl
ubap2a-MO (e3i3)
RT-
150 μM
300 μM
ubap2a
β-actin
aberrant transcript*
normal transcript
(344 bp)
β-actin (208 bp)
control MO
ubap2a MO (AUG)
d
α-Ubap2
e
α-Ubap2
3 dpf
f
***
Normalized
fluorescent intensity
2.0
1.5
1.0
cont MO
ubap2a MO
(AUG)
g
ATG
ubap2b
FP
111 bp
Exon 4
224 bp
145 bp
Exon 5
RP
ubap2b e4i4 MO
ubap2b i4e5 MO
h
cont MO: normal splicing
Exon 4
Exon 5
ubap2b MO (e4i4, i4e5) : intron inclusion
Exon 4
Exon 5
**intron inclusion: 222 bp insertion
leading to a premature stop codon
i
ubap2b MO
(e4i4)
bp
M
cont
600
800
(μM)
ubap2b
ef1α
aberrant transcript**
normal transcript
(256 bp)
ef1α (175 bp)
ubap2b MO
(i4e5)
bp
M
cont
600
800
(μM)
ubap2b
ef1α
aberrant transcript**
normal transcript
(256 bp)
ef1α (175 bp)

Supplementary Fig. 11. Construction of the splicing- and translation-blocking morpholinos (MOs) targeted to *ubap2a* and *ubap2b* genes in zebrafish. a A schematic diagram of the genomic structure of zebrafish *ubap2a* and the knockdown strategies. The morpholino target sites are shown as red rectangles targeting the splicing donor site of exon 3 (*ubap2a* e3i3 MO) and the translation start site in exon 2 (*ubap2a* AUG MO). Black arrows indicate PCR primers (FP, forward primer; RP, reverse primer) for reverse-transcription-PCR (RT-PCR). **b, c** A schematic diagram of the resulting transcripts of *ubap2a* after splicing (**b**) and results of RT-PCR (**c**) of *ubap2a* e3i3 MO-injected embryos. Normal transcript of the non-injected embryos (ctrl) and aberrant transcripts in the *ubap2a* e3i3 MO injected embryos at 3 dpf were confirmed using RT-PCR. RT-, no reverse transcriptase. *β-actin* was used as an RT-PCR control. **d, e** Whole-mount immunofluorescence of Ubap2 protein in the control MO-injected and *ubap2a* AUG MO (500 μM)-injected larvae at 3 dpf. Scale bar = 200 μm. **f** Quantification of Ubap2 fluorescence intensity in control MO (n = 7) and *ubap2a* AUG MO (n = 8) injected larvae. These values were measured using Image J, normalized by non-fluorescent background intensity. Statistical significance was determined using the two-tailed unpaired *t*-test. p = 0.0006. Data are presented as mean value ± SEM. **g** A schematic illustration of the genomic structure of zebrafish *ubap2b* and the knockdown strategies. The morpholino target sites are shown as red rectangles targeting the splicing donor site of exon 4 (*ubap2b* e4i4 MO) and the splicing acceptor of exon 5 (*ubap2b* i4e5 MO). Black arrows indicate PCR primers. **h, i** A schematic diagram (**h**) and RT-PCR analysis (**i**) showing efficacy of *ubap2b* e4i4 MO and *ubap2b* i4e5 MO in blocking normal splicing of *ubap2b*. *ef1α* is used as quality control for RNA integrity. Compared to control MO-injected larvae, aberrant transcripts (red arrows) are detected in *ubap2b* MO-injected larvae at 3 dpf. dpf, days post fertilization. Experiments in (c), (d), and (i) were independently repeated twice. Source data are provided as a Source Data file.

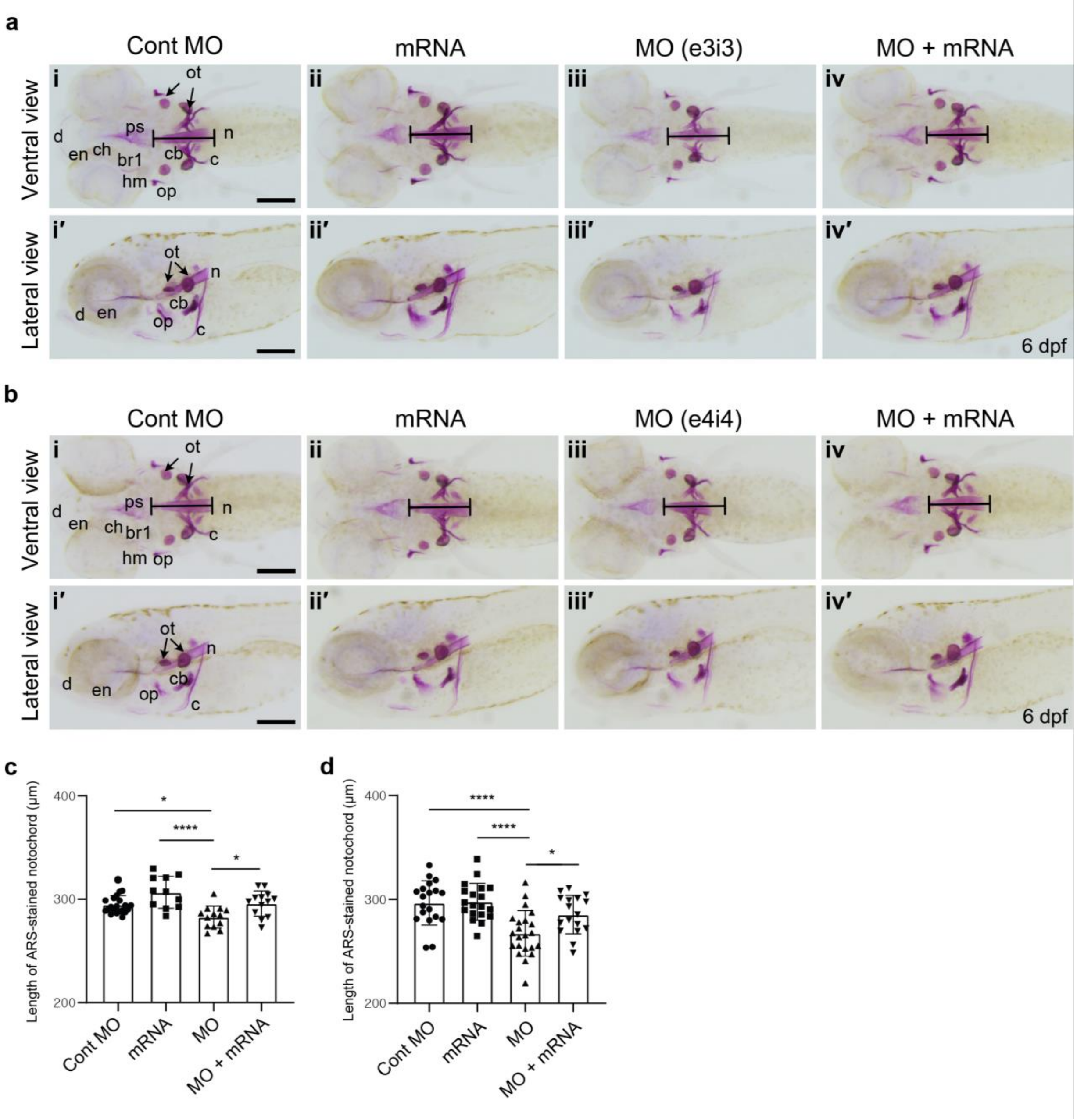

Supplementary Fig. 12. Rescue of the abnormal phenotypes of *ubap2a*- and *ubap2b*-knockdown zebrafishes during development by adding the depleted mRNAs. a Alizarin red S (ARS) staining of control morpholino (Cont MO) RNAs (**i, i′)**, *ubap2a* mRNA (**ii, ii′**), *ubap2a* MO (e3i3) (**iii, iii′**), and *ubap2a* MO+ *ubap2a* mRNA (**iv**, **iv′)**-injected larvae. **b** ARS staining of Cont MO RNAs (**i, i′)**, *ubap2b* mRNA (**ii, ii′**), *ubap2b* MO (e4i4) (**iii, iii′**), and *ubap2b* MO+ *ubap2b* mRNA (**iv**, **iv′)**-injected larvae. **c** Quantification of the length of ARS-stained notochord by ARS staining of zebrafish larvae injected with the control MO (Cont MO) (n = 21), *ubap2a* MO (e3i3) (n = 13), *ubap2a* mRNA (n = 11), and *ubap2a* MO plus *ubap2a* mRNA (n = 13). Statistical significance was determined using a one-way ANOVA with Tukey's test. All data are

presented as mean ± SD. **d** Quantification of the length of ARS-stained notochord by ARS staining of zebrafish larvae injected with the Cont MO (n = 19), *ubap2b* MO (e4i4) (n = 22), *ubap2b* mRNA (n = 19), and *ubap2b* MO plus *ubap2b* mRNA (n = 17). Concentrations of MOs: 800 μM for Cont MO, 300 μM for *ubap2a* e3i3 MO, and 800 μM for *ubap2b* e4i4 MO. Statistical significances were determined by ordinary one-way ANOVA with Tukey's test. *, $p < 0.05$ (Cont MO vs. MO, $p = 0.0239$; MO vs. MO + mRNA, $p = 0.0267$ for (c), and MO vs. MO + mRNA, $p = 0.0336$ for (d)) and ****, $p < 0.0001$ (for (c) and (d)). Data are presented as mean ± SEM. br1, branchiostegal ray1; c, cleithrum; cb, ceratobranchial 5; ch, ceratohyal; d, dentary; hm, hyomandibular; en, entopterygoid; m, maxilla; n, notochord; op, opercle; ot, otolith; and ps, parasphenoid. Scale bars = 200 μm. Source data are provided as a Source Data file.

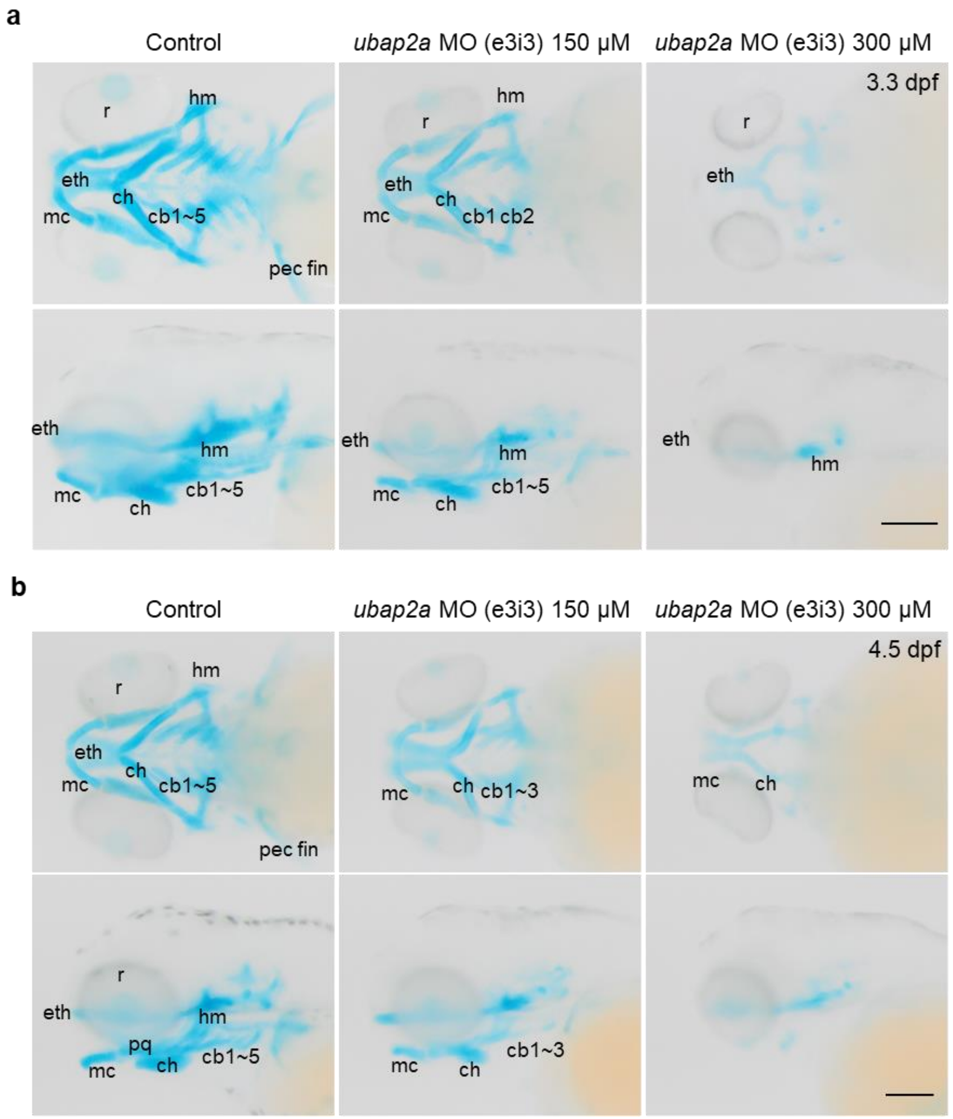

Supplementary Fig. 13. Phenotypes of ceratobranchial arche development in *ubap2*-knockdown zebrafish. a, b Alcian blue staining of *ubap2a*-morpholino (MO) RNA (e3i3)-injected zebrafish embryos at 3.3 days post fertilization (dpf) and 4.5 dpf. Fertilized eggs were injected with 150 µM or 300 µM of *ubap2*-MO RNA or not injected (Control). The cartilage structures of zebrafish larvae comprising craniofacial skeleton were visualized. Upper panels are ventral view and bottom panels are lateral view. Abbreviations: cb, ceratobranchial; ch, ceratohyal; eth, ethmoid plate; hm, hyomandibular; mc, Meckel's cartilage; pq, palatoquadrate; and r, retina. Scale bar = 100 µm. Experiments in (a) and (b) were independently repeated twice. Source data are provided as a Source Data file.

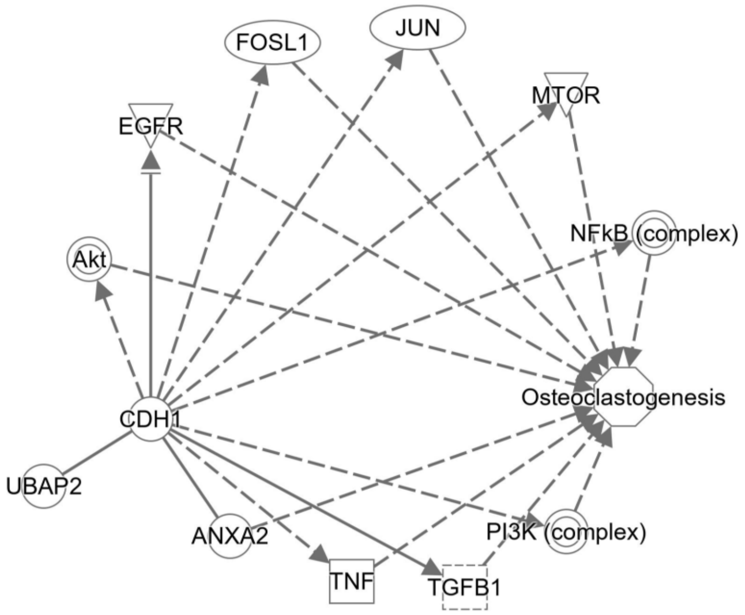

Supplementary Fig. 14. *In silico* network analysis between two key words 'UBAP2' and 'osteoclastogenesis'. Pathway network analysis between UBAP2 and osteoclastogenesis was conducted using the Path Explorer tool in Ingenuity Pathway Analysis (IPA) software (QIAGEN Inc., Hilden, Germany) (https://www.qiagenbioinformatics.com/products/ingenuity-pathway-analysis). The pathway networks of the connected molecules were formatted using IPA analysis with the QIAGEN Knowledge Base. Solid lines indicate direct interaction and dashed lines indicate indirect interaction.

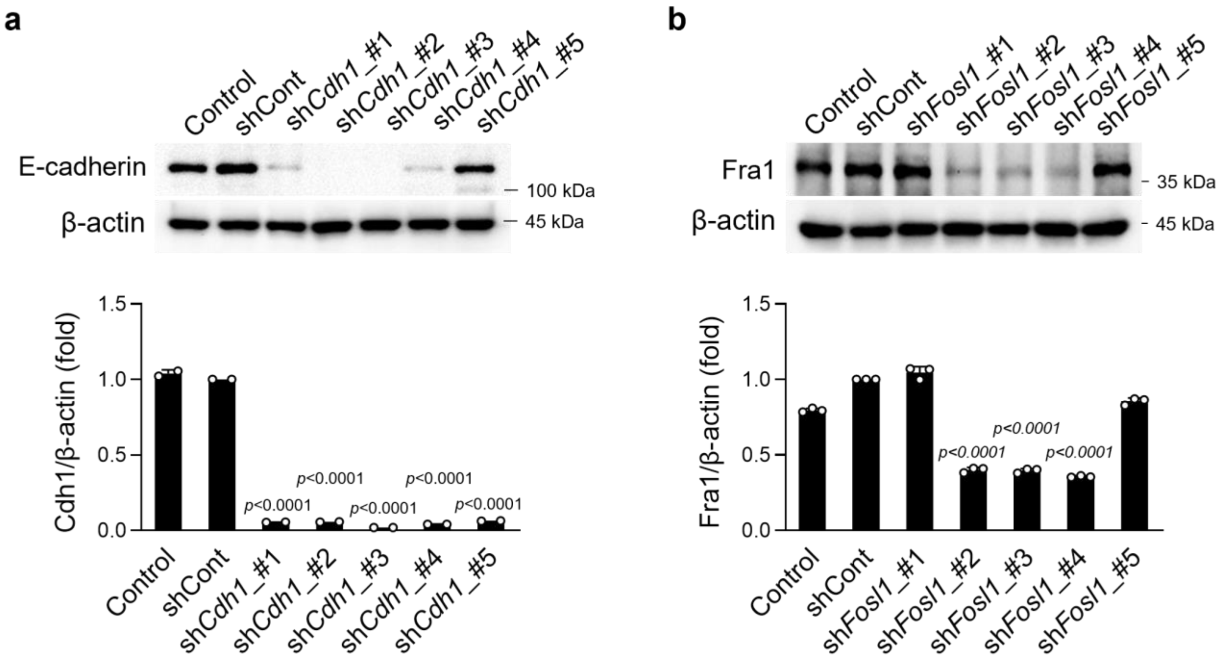

Supplementary Fig. 15. Evaluation of *Cdh1* and *Fosl1* knockdown constructs. Primary monocytes were infected with viral particles of pLKO.1-puro empty vector (shCont), **a)** *Cdh1* knockdown (sh*Cdh1*_#1, sh*Cdh1*_#2, sh*Cdh1*_#3, sh*Cdh1*_#4, and sh*Cdh1*_#5) constructs, and **b)** *Fosl1* knockdown (sh*Fosl1*_#1, sh *Fosl1*_#2, sh *Fosl1*_#3, sh *Fosl1*_#4, and sh *Fosl1*_#5) constructs. Immunoblotting was conducted with the anti-E-cadherin rabbit antibody, anti-Fra1 mouse antibody, and anti-β-actin mouse antibody. The intensity of protein bands for quantitative analysis was normalized to β-actin using Image Processing and Analysis in Java (Image J) software (http://imagej.nih.gov/ij/). All experiments were independently repeated twice. Statistical analyses were performed using Prism, version 9 (GraphPad) and statistical significance between the groups was calculated using the Student's *t*-test. Exact *p*-values representing comparison to short hairpin RNA control (shCont) are shown. All data are presented as mean ± SD with dot plots. Source data are provided as a Source Data file.

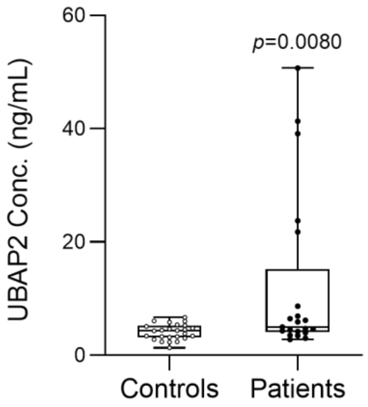

Supplementary Fig. 16. Replication analysis of UBAP2 protein concentration in blood plasma samples from normal controls and postmenopausal women with osteoporosis. To exclude the possibility of false positives of UBAP2 antibody, the levels of UBAP2 were re-measured in the blood plasma using an enzyme-linked immunosorbent assay (ELISA) kit of antibodies-online GmbH (Aachen, Germany). Blood plasmas were separated from whole peripheral blood samples from the normal control postmenopausal women (n = 13) and postmenopausal women with osteoporosis (n = 12). Protein concentrations (ng/ml) in plasmas were determined by analyzing the ELISA data. The concentrations were plotted with open and closed circles. The minimum to maximum concentrations and median values are shown in box-and-whisker plots. All experiments were repeated twice. Exact p-values of an unpaired two-tailed t-test are shown. Source data are provided as a Source Data file.

a

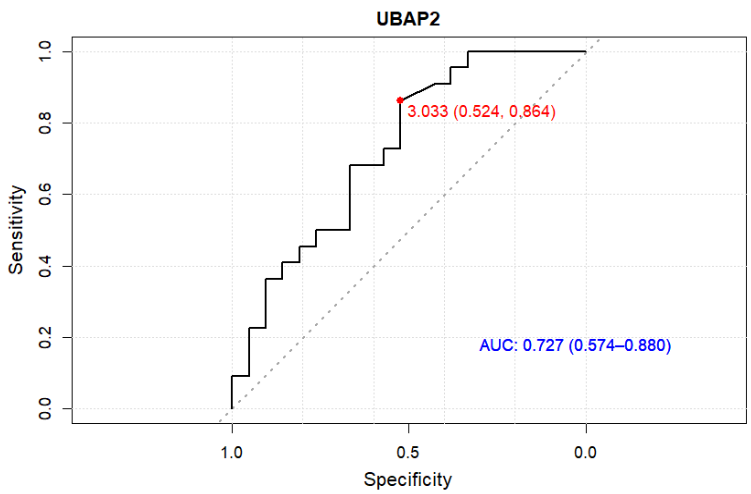

b

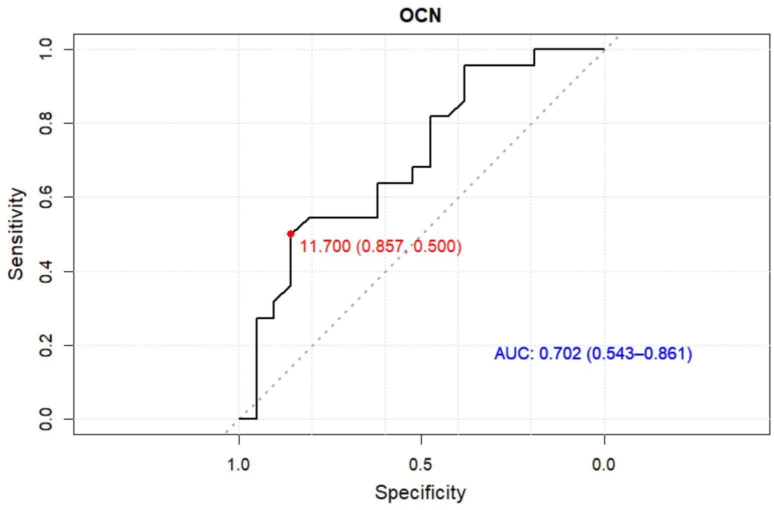

Supplementary Fig. 17. Results of receiver operating characteristic (ROC) curve analysis for ELISA values of UBAP2 and Osteocalcin (OCN). The ROC curve regression analysis was performed using R program of the pROC package (version 1.18)[2] with the ELISA value data of UBAP2 and OCN in Fig. 7. The optimal cut-off point, specificity, sensitivity, and the area under the curve (AUC) were determined. **a** Optimal cut-off point of UBAP2 was determined as 3.033 (specificities and sensitivities: 0.524, 0.864), and the AUC was 0.727 (CI: 0.574-0.880). **b** Optimal cut-off point of OCN was determined as 11.700 (specificities and sensitivities: 0.857, 0.500) and AUC was 0.702 (CI: 0.543-0.861).

Supplementary Table 1. Basic characteristics of the subjects participating in the case-control and quantitative trait exome-wide association studies in the Ansung and Ansan cohorts.

**Characteristics**	**Ansung**		**Ansan**		**Total**		
**Case-control analysis**	Case	Control	Case	Control	Case	Control	*p* -value*
Number of individuals	312	983	131	1,240	443	2,223	
Age (year)	60.92 ± 6.39	50.45 ± 7.91	59.06 ± 7.08	46.33 ± 6.04	60.37 ± 6.64	48.15 ± 7.23	< 0.01
Body mass indexs (BMI) (kg/m^2)	25.13 ± 3.49	24.44 ± 3.00	26.17 ± 3.19	24.22 ± 2.94	25.44 ± 3.43	24.32 ± 2.97	< 0.01
Distal radius speed of sound (DR-SOS) (m/s)	4027 ± 202	4277 ± 153	3908 ± 165	4269 ± 136	3991 ± 199	4272 ± 144	< 0.01
Midshaft tibia speed of sound (MT-SOS) (m/s)	3580 ± 99	3928 ± 104	3642 ± 127	3965 ± 119	3598 ± 112	3949 ± 114	< 0.01
DR-SOS T score	−1.26 ± 1.71	0.84 ± 1.28	−2.26 ± 1.43	0.77 ± 1.14	−1.56 ± 1.69	0.80 ± 1.20	< 0.01
MT-SOS T score	−3.53 ± 0.97	−0.18 ± 0.94	−2.91 ± 1.22	0.15 ± 1.08	−3.34 ± 1.08	0.002 ± 1.03	< 0.01
**Quantitative trait analysis**	Case	Control	Case	Control	Case	Control	*p* -value*
Number of individuals	1,861		1,708		3,569		
Age (year)	54.61 ± 8.92		48.63 ± 7.80		51.59 ± 8.89		
Body mass indexs (BMI) (kg/m^2)	24.74 ± 3.27		24.62 ± 3.17		24.68 ± 3.22		
Distal radius speed of sound (DR-SOS) (m/s)	4189 ± 192		4208 ± 179		4198 ± 186		
Midshaft tibia speed of sound (MT-SOS) (m/s)	3815 ± 164		3900 ± 158		3855 ± 167		
DR-SOS T score	0.10 ± 1.61		0.26 ± 1.50		0.18 ± 1.56		
MT-SOS T score	−1.27 ± 1.55		−0.47 ± 1.47		−0.89 ± 1.56		

*Significant differences in characteristics between the cases and controls were determined by two-tailed Student’s *t*-test.

Supplementary Table 2. List of top exonic SNPs suggestively associated with osteoporosis identified by exome-wide quantitative trait analysis for speed of sound T-score at midshaft tibia in the Korean women.

Chr	SNP	Gene	Consequence to transcript	A1	Discovery cohort (Ansung, *n* =1,861)		Replication cohort (Ansan, *n* =1,708)		Combined cohort (*n* =3,569)	
					beta ± se	Add *p*	beta ± se	Add *p*	beta ± se	Add *p*
9	rs2781	*UBAP2*	3′ UTR	C	−0.23 ±0.06	5.9×10^{-5}	−0.20 ±0.06	4.8×10^{-4}	−0.21 ±0.04	**1.1×10^{-7}**
12	rs9888363	*SYT10*	3′ UTR	A	−0.39 ±0.11	6.9×10^{-4}	−0.15 ±0.12	0.188	−0.28 ±0.08	7.1×10^{-4}
18	rs607230	*LAMA1*	Nonsynonymous (K2002E)	A	−0.19 ±0.06	7.8×10^{-4}	−0.03 ±0.06	0.642	−0.11 ±0.04	6.1×10^{-3}
12	rs479499	*CLEC12A*	Nonsynonymous (K244Q)	A	−0.26 ±0.08	8.1×10^{-4}	−0.07 ±0.08	0.078	−0.16 ±0.05	2.6×10^{-3}
5	rs2288395	*LOC100129870*	3′ UTR	G	0.15 ±0.05	9.6×10^{-4}	0.05 ±0.05	0.047	0.10 ±0.03	2.2×10^{-3}

Age and residential area were included as covariates in the additive genetic model. Abbreviations: A1, minor allele; Add *p*, additive model *p*-value; Chr, chromosome; se, standard error; SNP, single nucleotide polymorphism. The *p*-value below the Bonferroni-corrected significance level ($p < 7.7 \times 10^{-6}$) is indicated in bold. The SNP positions are based on the NCBI Build 36 human genome assembly.

Supplementary Table 3. Results of association analysis of the 96 SNPs (17 genotyped and 79 imputed* SNPs) in the *UBAP2* region associated with osteoporosis and bone density in the Korean women subjects.

SNP	A1	Funtion	Case-control analysis						Quantitative analysis for MT-SOS T-score						Quantitative analysis for DR-SOS T-score					
			Ansung cohort (314:983)		Ansan cohort (131:1,240)		Combined (2,223:443)		Ansung cohort (*n* =1,861)		Ansan cohort (*n* =1,708)		Combined (*n* =3,569)		Ansung cohort (*n* =1,824)		Ansan cohort (*n* =1,722)		Combined (*n* =3,546)	
			OR (95% CI)	Add *p*	OR (95% CI)	Add *p*	OR (95% CI)	Add *p*	beta ± se	Add *p*	beta ± se	Add *p*	beta ± se	Add *p*	beta ± se	Add *p*	beta ± se	Add *p*	beta ± se	Add *p*
rs7022524	T	down stream	1.20 (0.85−1.70)	0.296	1.06 (0.65−1.74)	0.815	1.17 (0.88−1.55)	0.286	−0.15 ±0.07	**0.044**	−0.24 ±0.08	**1.8.E-03**	−0.19 ±0.05	**3.5.E-04**	−0.07 ±0.08	0.370	−0.04 ±0.07	0.588	−0.06 ±0.05	0.260
rs4817	C	down stream	1.50 (1.14−1.99)	**4.3.E-03**	1.56 (1.06−2.27)	**0.023**	1.53 (1.23−1.92)	**1.9.E-04**	−0.21 ±0.06	**4.3.E-04**	−0.20 ±0.06	**8.2.E-04**	−0.20 ±0.04	**1.3.E-06**	−0.05 ±0.06	0.458	−0.06 ±0.06	0.285	−0.05 ±0.04	0.192
rs16935278	C	down stream	1.20 (0.85−1.70)	0.295	1.06 (0.65−1.74)	0.815	1.17 (0.88−1.55)	0.285	−0.15 ±0.07	**0.045**	−0.23 ±0.08	**1.9.E-03**	−0.19 ±0.05	**3.9.E-04**	−0.06 ±0.08	0.402	−0.04 ±0.07	0.572	−0.06 ±0.05	0.274
rs16935280	C	down stream	1.20 (0.85−1.70)	0.295	1.06 (0.65−1.74)	0.815	1.17 (0.88−1.55)	0.285	−0.15 ±0.07	**0.045**	−0.23 ±0.08	**1.9.E-03**	−0.19 ±0.05	**3.9.E-04**	−0.06 ±0.08	0.402	−0.04 ±0.07	0.572	−0.06 ±0.05	0.274
rs11848*	C	3′ UTR	0.88 (0.46−1.71)	0.715	1.42 (0.71−2.84)	0.319	1.08 (0.66−1.76)	0.751	0.10 ±0.15	0.495	−0.17 ±0.13	0.172	−0.05 ±0.10	0.574	0.05 ±0.15	0.738	−0.21 ±0.12	0.070	−0.11 ±0.09	0.251
rs2277178*	A	3′ UTR	1.03 (0.68−1.55)	0.889	1.26 (0.71−2.24)	0.427	1.10 (0.79−1.55)	0.562	−0.03 ±0.09	0.754	−0.22 ±0.09	**0.015**	−0.12 ±0.06	0.058	0.03 ±0.09	0.752	−0.13 ±0.08	0.122	−0.05 ±0.06	0.392
rs2781*	C	3′ UTR	1.64 (1.26−2.13)	**2.3.E-04**	1.85 (1.28−2.66)	**9.7.E-04**	1.72 (1.39−2.13)	**6.1.E-07**	−0.23 ±0.06	**5.2.E-05**	−0.20 ±0.06	**4.8.E-04**	−0.21 ±0.04	**1.1.E-07**	−0.03 ±0.06	0.645	−0.11 ±0.05	**0.040**	−0.07 ±0.04	0.076
rs2296365	C	intron	1.20 (0.85−1.70)	0.296	1.07 (0.66−1.76)	0.778	1.17 (0.88−1.55)	0.274	−0.15 ±0.07	**0.045**	−0.23 ±0.08	**1.9.E-03**	−0.19 ±0.05	**4.0.E-04**	−0.06 ±0.08	0.409	−0.04 ±0.07	0.539	−0.06 ±0.05	0.264
rs307695	G	intron	1.20 (0.85−1.70)	0.296	1.07 (0.66−1.76)	0.778	1.17 (0.88−1.55)	0.274	−0.15 ±0.07	**0.044**	−0.23 ±0.08	**1.9.E-03**	−0.19 ±0.05	**3.8.E-04**	−0.06 ±0.08	0.397	−0.04 ±0.07	0.538	−0.06 ±0.05	0.257
rs307694	A	intron	1.56 (1.19−2.04)	**1.3.E-03**	1.74 (1.20−2.53)	**3.4.E-03**	1.63 (1.31−2.03)	**1.1.E-05**	−0.22 ±0.06	**1.6.E-04**	−0.21 ±0.06	**5.1.E-04**	−0.21 ±0.04	**3.2.E-07**	−0.04 ±0.06	0.561	−0.07 ±0.06	0.225	−0.05 ±0.04	0.198
rs10971796	A	intron	1.84 (1.27−2.66)	**1.4.E-03**	2.38 (1.45−3.93)	**6.6.E-04**	2.02 (1.50−2.72)	**4.2.E-06**	−0.23 ±0.08	**4.5.E-03**	−0.11 ±0.08	0.202	−0.17 ±0.06	**3.1.E-03**	0.02 ±0.09	0.770	−0.07 ±0.08	0.403	−0.02 ±0.06	0.771
rs307693	A	intron	1.20 (0.85−1.70)	0.296	1.07 (0.66−1.76)	0.778	1.17 (0.88−1.55)	0.274	−0.15 ±0.07	**4.4.E-02**	−0.23 ±0.08	**1.9.E-03**	−0.19 ±0.05	**3.8.E-04**	−0.06 ±0.08	0.397	−0.04 ±0.07	0.538	−0.06 ±0.05	0.257
rs745532	A	intron	1.56 (1.19−2.04)	**1.3.E-03**	1.74 (1.20−2.53)	**3.4.E-03**	1.63 (1.31−2.03)	**1.1.E-05**	−0.22 ±0.06	**1.6.E-04**	−0.21 ±0.06	**5.1.E-04**	−0.21 ±0.04	**3.2.E-07**	−0.04 ±0.06	0.561	−0.07 ±0.06	0.225	−0.05 ±0.04	0.198
rs307691	C	intron	1.56 (1.19−2.04)	**1.4.E-03**	1.74 (1.20−2.53)	**3.4.E-03**	1.63 (1.31−2.03)	**1.1.E-05**	−0.22 ±0.06	**1.9.E-04**	−0.21 ±0.06	**5.1.E-04**	−0.21 ±0.04	**3.8.E-07**	−0.03 ±0.06	0.569	−0.07 ±0.06	0.225	−0.05 ±0.04	0.202
rs307690	T	intron	1.20 (0.85−1.70)	0.296	1.07 (0.66−1.76)	0.778	1.17 (0.88−1.55)	0.274	−0.15 ±0.07	**0.044**	−0.23 ±0.08	**2.0.E-03**	−0.19 ±0.05	**3.9.E-04**	−0.06 ±0.08	0.397	−0.04 ±0.07	0.535	−0.06 ±0.05	0.256
rs307689	A	intron	1.20 (0.85−1.70)	0.296	1.07 (0.66−1.76)	0.778	1.17 (0.88−1.55)	0.274	−0.15 ±0.07	**0.044**	−0.23 ±0.08	**2.0.E-03**	−0.19 ±0.05	**3.9.E-04**	−0.06 ±0.08	0.397	−0.04 ±0.07	0.535	−0.06 ±0.05	0.256
rs10120819	C	intron	1.84 (1.27−2.66)	**1.4.E-03**	2.38 (1.45−3.93)	**6.6.E-04**	2.02 (1.50−2.72)	**4.1.E-06**	−0.23 ±0.08	**4.6.E-03**	−0.11 ±0.08	0.202	−0.17 ±0.06	**3.2.E-03**	0.02 ±0.09	0.770	−0.07 ±0.08	0.403	−0.02 ±0.06	0.771
rs307688	G	intron	1.56 (1.19−2.04)	**1.4.E-03**	1.74 (1.20−2.53)	**3.4.E-03**	1.63 (1.31−2.03)	**1.1.E-05**	−0.22 ±0.06	**1.9.E-04**	−0.20 ±0.06	**5.4.E-04**	−0.21 ±0.04	**4.0.E-07**	−0.03 ±0.06	0.569	−0.07 ±0.06	0.222	−0.05 ±0.04	0.200
rs307687*	C	intron	1.20 (0.86−1.68)	0.276	1.10 (0.68−1.79)	0.702	1.18 (0.90−1.55)	0.240	−0.17 ±0.07	**0.019**	−0.14 ±0.07	**0.048**	−0.15 ±0.05	**2.4.E-03**	−0.07 ±0.07	0.317	0.02 ±0.07	0.738	−0.03 ±0.05	0.548
rs307642	T	intron	1.56 (1.19−2.04)	**1.4.E-03**	1.74 (1.20−2.53)	**3.4.E-03**	1.63 (1.31−2.03)	**1.1.E-05**	−0.22 ±0.06	**1.9.E-04**	−0.20 ±0.06	**5.4.E-04**	−0.21 ±0.04	**4.0.E-07**	−0.03 ±0.06	0.569	−0.07 ±0.06	0.222	−0.05 ±0.04	0.200
rs307645	A	intron	1.56 (1.19−2.04)	**1.4.E-03**	1.74 (1.20−2.53)	**3.4.E-03**	1.63 (1.31−2.03)	**1.1.E-05**	−0.22 ±0.06	**1.9.E-04**	−0.20 ±0.06	**5.4.E-04**	−0.21 ±0.04	**4.0.E-07**	−0.03 ±0.06	0.569	−0.07 ±0.06	0.222	−0.05 ±0.04	0.200
rs307646*	G	intron	1.59 (1.22−2.08)	**6.1.E-04**	1.74 (1.21−2.52)	**3.2.E-03**	1.66 (1.34−2.06)	**4.3.E-06**	−0.22 ±0.06	**9.8.E-05**	−0.19 ±0.06	**9.0.E-04**	−0.21 ±0.04	**3.5.E-07**	−0.02 ±0.06	0.674	−0.09 ±0.05	0.104	−0.06 ±0.04	0.149
rs307647	G	intron	1.56 (1.19−2.04)	**1.4.E-03**	1.74 (1.20−2.53)	**3.4.E-03**	1.63 (1.31−2.03)	**1.1.E-05**	−0.22 ±0.06	**1.9.E-04**	−0.20 ±0.06	**5.4.E-04**	−0.21 ±0.04	**4.0.E-07**	−0.03 ±0.06	0.569	−0.07 ±0.06	0.222	−0.05 ±0.04	0.200
rs307648	C	intron	1.56 (1.19−2.04)	**1.4.E-03**	1.74 (1.20−2.53)	**3.4.E-03**	1.63 (1.31−2.03)	**1.1.E-05**	−0.22 ±0.06	**1.9.E-04**	−0.20 ±0.06	**5.4.E-04**	−0.21 ±0.04	**4.0.E-07**	−0.03 ±0.06	0.569	−0.07 ±0.06	0.222	−0.05 ±0.04	0.200
rs307649	T	intron	1.51 (1.14−2.00)	**4.1.E-03**	1.55 (1.06−2.27)	**0.024**	1.54 (1.23−1.93)	**1.9.E-04**	−0.22 ±0.06	**2.7.E-04**	−0.19 ±0.06	**1.5.E-03**	−0.20 ±0.04	**1.5.E-06**	−0.06 ±0.06	0.353	−0.06 ±0.06	0.318	−0.06 ±0.04	0.164
rs874572*	G	intron	1.80 (1.21−2.68)	**3.6.E-03**	2.24 (1.33−3.77)	**2.4.E-03**	1.95 (1.42−2.67)	**3.2.E-05**	−0.24 ±0.09	**5.8.E-03**	−0.11 ±0.08	0.207	−0.17 ±0.06	**4.2.E-03**	0.00 ±0.09	0.985	−0.08 ±0.08	0.333	−0.04 ±0.06	0.536
rs307650	A	intron	1.20 (0.85−1.70)	0.296	1.07 (0.66−1.76)	0.778	1.17 (0.88−1.55)	0.274	−0.15 ±0.07	**0.044**	−0.23 ±0.08	**2.0.E-03**	−0.19 ±0.05	**3.9.E-04**	−0.06 ±0.08	0.397	−0.04 ±0.07	0.535	−0.06 ±0.05	0.256
rs12377078*	A	intron	1.95 (0.71−5.36)	0.195	2.77 (0.62−12.25)	0.181	2.20 (0.96−5.01)	0.062	−0.28 ±0.29	0.338	−0.20 ±0.26	0.452	−0.23 ±0.19	0.235	−0.04 ±0.30	0.897	−0.42 ±0.24	0.079	−0.26 ±0.19	0.167
rs307651	A	intron	1.50 (1.14−1.99)	**4.4.E-03**	1.53 (1.05−2.24)	**0.028**	1.53 (1.22−1.91)	**2.3.E-04**	−0.21 ±0.06	**4.2.E-04**	−0.19 ±0.06	**1.1.E-03**	−0.20 ±0.04	**1.7.E-06**	−0.04 ±0.06	0.475	−0.06 ±0.06	0.266	−0.05 ±0.04	0.189
rs307652*	A	intron	1.12 (0.87−1.43)	0.374	0.92 (0.63−1.35)	0.686	1.06 (0.86−1.31)	0.564	0.01 ±0.05	0.833	0.06 ±0.06	0.255	0.04 ±0.04	0.351	−0.07 ±0.05	0.219	0.01 ±0.05	0.877	−0.03 ±0.04	0.402
rs307657	C	intron	1.50 (1.14−1.99)	**4.4.E-03**	1.53 (1.05−2.24)	**0.028**	1.53 (1.22−1.91)	**2.3.E-04**	−0.21 ±0.06	**4.2.E-04**	−0.19 ±0.06	**1.1.E-03**	−0.20 ±0.04	**1.7.E-06**	−0.04 ±0.06	0.475	−0.06 ±0.06	0.266	−0.05 ±0.04	0.189
rs307658	C	missense (N606S)	1.50 (1.14−1.99)	**4.4.E-03**	1.53 (1.05−2.24)	**0.028**	1.53 (1.22−1.91)	**2.3.E-04**	−0.21 ±0.06	**4.2.E-04**	−0.19 ±0.06	**1.1.E-03**	−0.20 ±0.04	**1.7.E-06**	−0.04 ±0.06	0.475	−0.06 ±0.06	0.266	−0.05 ±0.04	0.189
rs307660	C	intron	1.14 (0.89−1.47)	0.292	0.96 (0.65−1.40)	0.822	1.09 (0.89−1.34)	0.414	0.01 ±0.05	0.840	0.05 ±0.06	0.364	0.03 ±0.04	0.441	−0.06 ±0.05	0.277	0.01 ±0.05	0.918	−0.03 ±0.04	0.447
rs307654	T	intron	1.15 (0.89−1.50)	0.289	0.98 (0.66−1.45)	0.929	1.10 (0.89−1.37)	0.379	−0.03 ±0.06	0.542	0.04 ±0.06	0.459	0.00 ±0.04	0.956	−0.05 ±0.06	0.391	0.05 ±0.06	0.354	0.00 ±0.04	0.966
rs595556	C	intron	1.50 (1.14−1.99)	**4.4.E-03**	1.53 (1.05−2.24)	**0.028**	1.53 (1.22−1.91)	**2.3.E-04**	−0.21 ±0.06	**4.2.E-04**	−0.19 ±0.06	**1.1.E-03**	−0.20 ±0.04	**1.7.E-06**	−0.04 ±0.06	0.475	−0.06 ±0.06	0.266	−0.05 ±0.04	0.189
rs10971818*	A	intron	1.56 (0.72−3.40)	0.261	3.44 (1.53−7.73)	**2.8.E-03**	2.20 (1.25−3.87)	**6.0.E-03**	−0.27 ±0.21	0.198	−0.27 ±0.18	0.133	−0.27 ±0.14	**0.050**	−0.04 ±0.21	0.841	−0.41 ±0.17	**0.016**	−0.26 ±0.13	0.057
rs16935299	T	intron	1.42 (1.14−1.77)	**2.0.E-03**	1.32 (0.96−1.81)	0.083	1.40 (1.17−1.68)	**2.8.E-04**	−0.15 ±0.05	**1.1.E-03**	−0.09 ±0.05	**0.043**	−0.12 ±0.03	**1.8.E-04**	−0.06 ±0.05	0.191	−0.01 ±0.04	0.869	−0.04 ±0.03	0.267
rs558415	C	intron	1.20 (0.85−1.70)	0.296	1.07 (0.66−1.76)	0.778	1.17 (0.88−1.55)	0.274	−0.15 ±0.07	**0.044**	−0.23 ±0.08	**2.0.E-03**	−0.19 ±0.05	**3.9.E-04**	−0.06 ±0.08	0.397	−0.04 ±0.07	0.535	−0.06 ±0.05	0.256
rs544169	G	intron	1.50 (1.14−1.99)	**4.3.E-03**	1.56 (1.07−2.27)	**0.022**	1.54 (1.23−1.92)	**1.9.E-04**	−0.21 ±0.06	**4.5.E-04**	−0.20 ±0.06	**8.0.E-04**	−0.20 ±0.04	**1.3.E-06**	−0.04 ±0.06	0.497	−0.06 ±0.06	0.246	−0.05 ±0.04	0.187
rs668071	C	intron	1.50 (1.14−1.99)	**4.3.E-03**	1.56 (1.07−2.27)	**0.022**	1.54 (1.23−1.92)	**1.9.E-04**	−0.21 ±0.06	**4.5.E-04**	−0.20 ±0.06	**8.0.E-04**	−0.20 ±0.04	**1.3.E-06**	−0.04 ±0.06	0.497	−0.06 ±0.06	0.246	−0.05 ±0.04	0.187
rs10971822	A	intron	1.80 (1.21−2.68)	**3.6.E-03**	2.24 (1.33−3.78)	**2.4.E-03**	1.95 (1.42−2.67)	**3.2.E-05**	−0.24 ±0.09	**5.8.E-03**	−0.12 ±0.08	0.165	−0.18 ±0.06	**3.2.E-03**	0.00 ±0.09	0.985	−0.08 ±0.08	0.321	−0.04 ±0.06	0.524
rs307661	T	intron	1.18 (0.92−1.53)	0.198	1.06 (0.72−1.54)	0.773	1.15 (0.93−1.42)	0.197	−0.04 ±0.06	0.448	0.03 ±0.06	0.592	−0.01 ±0.04	0.851	−0.05 ±0.06	0.348	0.04 ±0.05	0.521	−0.01 ±0.04	0.761
rs307663	G	intron	1.20 (0.85−1.70)	0.294	1.06 (0.65−1.74)	0.815	1.17 (0.88−1.55)	0.285	−0.15 ±0.07	**0.045**	−0.23 ±0.08	**2.3.E-03**	−0.19 ±0.05	**4.5.E-04**	−0.06 ±0.08	0.412	−0.04 ±0.07	0.550	−0.06 ±0.05	0.270
rs307665	G	intron	1.20 (0.85−1.70)	0.294	1.06 (0.65−1.74)	0.815	1.17 (0.88−1.55)	0.285	−0.15 ±0.07	**0.045**	−0.23 ±0.08	**2.3.E-03**	−0.19 ±0.05	**4.5.E-04**	−0.06 ±0.08	0.412	−0.04 ±0.07	0.550	−0.06 ±0.05	0.270
rs307670	T	intron	1.50 (1.14−1.99)	**4.3.E-03**	1.56 (1.07−2.27)	**0.022**	1.54 (1.23−1.92)	**1.9.E-04**	−0.21 ±0.06	**4.5.E-04**	−0.20 ±0.06	**8.0.E-04**	−0.20 ±0.04	**1.3.E-06**	−0.04 ±0.06	0.497	−0.06 ±0.06	0.246	−0.05 ±0.04	0.187
rs307678	A	intron	1.50 (1.14−1.99)	**4.3.E-03**	1.56 (1.07−2.27)	**0.022**	1.54 (1.23−1.92)	**1.9.E-04**	−0.21 ±0.06	**4.5.E-04**	−0.20 ±0.06	**8.0.E-04**	−0.20 ±0.04	**1.3.E-06**	−0.04 ±0.06	0.497	−0.06 ±0.06	0.246	−0.05 ±0.04	0.187
rs307679	C	intron	1.20 (0.85−1.70)	0.294	1.06 (0.65−1.74)	0.815	1.17 (0.88−1.55)	0.285	−0.15 ±0.07	**0.045**	−0.23 ±0.08	**2.3.E-03**	−0.19 ±0.05	**4.5.E-04**	−0.06 ±0.08	0.412	−0.04 ±0.07	0.550	−0.06 ±0.05	0.270
rs307680	C	intron	1.20 (0.85−1.70)	0.294	1.06 (0.65−1.74)	0.815	1.17 (0.88−1.55)	0.285	−0.15 ±0.07	**0.045**	−0.23 ±0.08	**2.3.E-03**	−0.19 ±0.05	**4.5.E-04**	−0.06 ±0.08	0.412	−0.04 ±0.07	0.550	−0.06 ±0.05	0.270
rs3739690	A	synonymous (P196P)	1.43 (1.15−1.79)	**1.4.E-03**	1.39 (1.01−1.90)	**0.042**	1.43 (1.20−1.72)	**9.8.E-05**	−0.15 ±0.05	**1.0.E-03**	−0.11 ±0.05	**0.021**	−0.13 ±0.03	**7.7.E-05**	−0.06 ±0.05	0.207	−0.02 ±0.04	0.641	−0.04 ±0.03	0.197
rs307681	A	intron	1.20 (0.85−1.70)	0.294	1.06 (0.65−1.74)	0.815	1.17 (0.88−1.55)	0.285	−0.15 ±0.07	**0.045**	−0.23 ±0.08	**2.3.E-03**	−0.19 ±0.05	**4.5.E-04**	−0.06 ±0.08	0.412	−0.04 ±0.07	0.550	−0.06 ±0.05	0.270
rs307682*	G	intron	1.49 (1.12−1.97)	**6.0.E-03**	1.56 (1.07−2.29)	**0.022**	1.53 (1.22−1.91)	**2.6.E-04**	−0.20 ±0.06	**9.4.E-04**	−0.20 ±0.06	**7.3.E-04**	−0.20 ±0.04	**2.5.E-06**	−0.03 ±0.06	0.606	−0.07 ±0.06	0.210	−0.05 ±0.04	0.206
rs307683	C	intron	1.50 (1.14−1.99)	**4.3.E-03**	1.56 (1.07−2.27)	**0.022**	1.54 (1.23−1.92)	**1.9.E-04**	−0.21 ±0.06	**4.5.E-04**	−0.20 ±0.06	**8.0.E-04**	−0.20 ±0.04	**1.3.E-06**	−0.04 ±0.06	0.497	−0.06 ±0.06	0.246	−0.05 ±0.04	0.187
rs4272470	G	intron	1.50 (1.14−1.99)	**4.3.E-03**	1.56 (1.07−2.27)	**0.022**	1.54 (1.23−1.92)	**1.9.E-04**	−0.21 ±0.06	**4.5.E-04**	−0.20 ±0.06	**8.0.E-04**	−0.20 ±0.04	**1.3.E-06**	−0.04 ±0.06	0.497	−0.06 ±0.06	0.246	−0.05 ±0.04	0.187
rs307697	C	intron	1.50 (1.14−1.99)	**4.3.E-03**	1.56 (1.07−2.27)	**0.022**	1.54 (1.23−1.92)	**1.9.E-04**	−0.21 ±0.06	**4.5.E-04**	−0.20 ±0.06	**8.0.E-04**	−0.20 ±0.04	**1.3.E-06**	−0.04 ±0.06	0.497	−0.06 ±0.06	0.246	−0.05 ±0.04	0.187
rs307699*	T	intron	1.20 (0.85−1.70)	0.294	1.02 (0.62−1.67)	0.932	1.15 (0.87−1.53)	0.324	−0.15 ±0.07	**0.045**	−0.22 ±0.08	**3.4.E-03**	−0.18 ±0.05	**6.0.E-04**	−0.06 ±0.08	0.412	−0.03 ±0.07	0.650	−0.05 ±0.05	0.312
rs307700	G	intron	1.50 (1.14−1.99)	**4.3.E-03**	1.56 (1.07−2.27)	**0.022**	1.54 (1.23−1.92)	**1.9.E-04**	−0.21 ±0.06	**4.5.E-04**	−0.20 ±0.06	**8.0.E-04**	−0.20 ±0.04	**1.3.E-06**	−0.04 ±0.06	0.497	−0.06 ±0.06	0.246	−0.05 ±0.04	0.187
rs307703	G	intron	1.17 (0.92−1.50)	0.202	1.05 (0.72−1.52)	0.814	1.14 (0.93−1.40)	0.205	0.00 ±0.05	0.969	0.04 ±0.06	0.428	0.02 ±0.04	0.603	−0.07 ±0.05	0.195	−0.01 ±0.05	0.891	−0.04 ±0.04	0.276
rs834027	T	intron	1.50 (1.14−1.99)	**4.3.E-03**	1.56 (1.07−2.27)	**0.022**	1.54 (1.23−1.92)	**1.9.E-04**	−0.21 ±0.06	**4.5.E-04**	−0.20 ±0.06	**8.0.E-04**	−0.20 ±0.04	**1.3.E-06**	−0.04 ±0.06	0.497	−0.06 ±0.06	0.246	−0.05 ±0.04	0.187
rs10971836	C	intron	1.43 (1.14−1.78)	**1.8.E-03**	1.41 (1.03−1.93)	**0.035**	1.43 (1.20−1.72)	**1.0.E-04**	−0.15 ±0.05	**1.2.E-03**	−0.11 ±0.05	**0.019**	−0.13 ±0.03	**8.0.E-05**	−0.07 ±0.05	0.173	−0.03 ±0.04	0.530	−0.05 ±0.03	0.141
rs6476429	A	intron	1.50 (1.14−1.99)	**4.3.E-03**	1.56 (1.07−2.27)	**0.022**	1.54 (1.23−1.92)	**1.9.E-04**	−0.21 ±0.06	**4.5.E-04**	−0.20 ±0.06	**8.0.E-04**	−0.20 ±0.04	**1.3.E-06**	−0.04 ±0.06	0.497	−0.06 ±0.06	0.246	−0.05 ±0.04	0.187
rs10814058	G	intron	1.50 (1.14−1.99)	**4.3.E-03**	1.56 (1.07−2.27)	**0.022**	1.54 (1.23−1.92)	**1.9.E-04**	−0.21 ±0.06	**4.5.E-04**	−0.20 ±0.06	**8.0.E-04**	−0.20 ±0.04	**1.3.E-06**	−0.04 ±0.06	0.497	−0.06 ±0.06	0.246	−0.05 ±0.04	0.187
rs932969	C	intron	1.80 (1.21−2.68)	**3.6.E-03**	2.24 (1.33−3.78)	**0.002**	1.95 (1.42−2.67)	**3.2.E-05**	−0.24 ±0.09	**5.8.E-03**	−0.12 ±0.08	0.165	−0.18 ±0.06	**3.2.E-03**	0.00 ±0.09	0.985	−0.08 ±0.08	0.321	−0.04 ±0.06	0.524
rs10814059	T	intron	1.50 (1.14−1.99)	**4.3.E-03**	1.56 (1.07−2.27)	**0.022**	1.54 (1.23−1.92)	**1.9.E-04**	−0.21 ±0.06	**4.5.E-04**	−0.20 ±0.06	**8.0.E-04**	−0.20 ±0.04	**1.3.E-06**	−0.04 ±0.06	0.497	−0.06 ±0.06	0.246	−0.05 ±0.04	0.187
rs10814060	G	intron	1.50 (1.14−1.99)	**4.3.E-03**	1.56 (1.07−2.27)	**0.022**	1.54 (1.23−1.92)	**1.9.E-04**	−0.21 ±0.06	**4.5.E-04**	−0.20 ±0.06	**8.0.E-04**	−0.20 ±0.04	**1.3.E-06**	−0.04 ±0.06	0.497	−0.06 ±0.06	0.246	−0.05 ±0.04	0.187
rs12555291	A	intron	1.18 (0.92−1.53)	0.198	1.06 (0.72−1.54)	0.773	1.15 (0.93−1.42)	0.197	−0.04 ±0.06	0.448	0.03 ±0.06	0.592	−0.01 ±0.04	0.851	−0.05 ±0.06	0.348	0.04 ±0.05	0.521	−0.01 ±0.04	0.761
rs7029112	C	intron	1.50 (1.14−1.99)	**4.3.E-03**	1.56 (1.07−2.27)	**0.022**	1.54 (1.23−1.92)	**1.9.E-04**	−0.21 ±0.06	**4.5.E-04**	−0.20 ±0.06	**8.0.E-04**	−0.20 ±0.04	**1.3.E-06**	−0.04 ±0.06	0.497	−0.06 ±0.06	0.246	−0.05 ±0.04	0.187
rs7029122	C	intron	1.20 (0.85−1.70)	0.294	1.06 (0.65−1.74)	0.815	1.17 (0.88−1.55)	0.285	−0.15 ±0.07	**0.045**	−0.23 ±0.08	**2.3.E-03**	−0.19 ±0.05	**4.5.E-04**	−0.06 ±0.08	0.412	−0.04 ±0.07	0.550	−0.06 ±0.05	0.270
rs10465080	T	intron	1.42 (1.14−1.77)	**1.7.E-03**	1.39 (1.01−1.91)	**0.047**	1.43 (1.19−1.71)	**1.3.E-04**	−0.12 ±0.05	**8.8.E-03**	−0.10 ±0.05	**0.030**	−0.11 ±0.03	**7.5.E-04**	−0.08 ±0.05	0.096	−0.05 ±0.04	0.227	−0.07 ±0.03	**0.037**
rs10758236	C	intron	1.50 (1.14−1.99)	**4.3.E-03**	1.56 (1.07−2.27)	**0.022**	1.54 (1.23−1.92)	**1.9.E-04**	−0.21 ±0.06	**4.5.E-04**	−0.20 ±0.06	**8.0.E-04**	−0.20 ±0.04	**1.3.E-06**	−0.04 ±0.06	0.497	−0.06 ±0.06	0.246	−0.05 ±0.04	0.187
rs3016756	C	intron	1.43 (1.15−1.79)	**1.4.E-03**	1.39 (1.01−1.90)	**0.042**	1.43 (1.20−1.72)	**9.8.E-05**	−0.15 ±0.05	**1.0.E-03**	−0.11 ±0.05	**0.021**	−0.13 ±0.03	**7.7.E-05**	−0.06 ±0.05	0.207	−0.02 ±0.04	0.641	−0.04 ±0.03	0.197
rs1785506	C	missense (R154Q)	1.50 (1.14−1.99)	**4.3.E-03**	1.56 (1.07−2.27)	**0.022**	1.54 (1.23−1.92)	**1.9.E-04**	−0.21 ±0.06	**4.5.E-04**	−0.20 ±0.06	**8.0.E-04**	−0.20 ±0.04	**1.3.E-06**	−0.04 ±0.06	0.497	−0.06 ±0.06	0.246	−0.05 ±0.04	0.187
rs1785503	C	intron	1.50 (1.14−1.99)	**4.3.E-03**	1.56 (1.07−2.27)	**0.022**	1.54 (1.23−1.92)	**1.9.E-04**	−0.21 ±0.06	**4.5.E-04**	−0.20 ±0.06	**8.0.E-04**	−0.20 ±0.04	**1.3.E-06**	−0.04 ±0.06	0.497	−0.06 ±0.06	0.246	−0.05 ±0.04	0.187
rs12553933	G	intron	1.18 (0.92−1.53)	0.198	1.06 (0.72−1.54)	0.773	1.15 (0.93−1.42)	0.197	−0.04 ±0.06	0.448	0.03 ±0.06	0.592	−0.01 ±0.04	0.851	−0.05 ±0.06	0.348	0.04 ±0.05	0.521	−0.01 ±0.04	0.761
rs12164256	G	intron	1.42 (1.14−1.77)	**1.7.E-03**	1.39 (1.01−1.91)	**0.047**	1.43 (1.19−1.71)	**1.3.E-04**	−0.12 ±0.05	**8.8.E-03**	−0.10 ±0.05	**0.030**	−0.11 ±0.03	**7.5.E-04**	−0.08 ±0.05	0.096	−0.05 ±0.04	0.227	−0.07 ±0.03	**0.037**
rs1785502	C	intron	1.50 (1.14−1.99)	**4.3.E-03**	1.56 (1.07−2.27)	**0.022**	1.54 (1.23−1.92)	**1.9.E-04**	−0.21 ±0.06	**4.5.E-04**	−0.20 ±0.06	**8.0.E-04**	−0.20 ±0.04	**1.3.E-06**	−0.04 ±0.06	0.497	−0.06 ±0.06	0.246	−0.05 ±0.04	0.187
rs1785500	C	intron	1.50 (1.14−1.99)	**4.3.E-03**	1.56 (1.07−2.27)	**0.022**	1.54 (1.23−1.92)	**1.9.E-04**	−0.21 ±0.06	**4.5.E-04**	−0.20 ±0.06	**8.0.E-04**	−0.20 ±0.04	**1.3.E-06**	−0.04 ±0.06	0.497	−0.06 ±0.06	0.246	−0.05 ±0.04	0.187
rs1758632	C	intron	1.50 (1.14−1.99)	**4.3.E-03**	1.56 (1.07−2.27)	**0.022**	1.54 (1.23−1.92)	**1.9.E-04**	−0.21 ±0.06	**4.5.E-04**	−0.20 ±0.06	**8.0.E-04**	−0.20 ±0.04	**1.3.E-06**	−0.04 ±0.06	0.497	−0.06 ±0.06	0.246	−0.05 ±0.04	0.187
rs1785512	C	intron	1.50 (1.14−1.99)	**4.3.E-03**	1.56 (1.07−2.27)	**0.022**	1.54 (1.23−1.92)	**1.9.E-04**	−0.21 ±0.06	**4.5.E-04**	−0.20 ±0.06	**8.0.E-04**	−0.20 ±0.04	**1.3.E-06**	−0.04 ±0.06	0.497	−0.06 ±0.06	0.246	−0.05 ±0.04	0.187
rs1758633*	C	intron	1.50 (1.13−1.98)	**4.4.E-03**	1.57 (1.07−2.29)	**0.020**	1.54 (1.23−1.92)	**1.8.E-04**	−0.21 ±0.06	**4.4.E-04**	−0.20 ±0.06	**6.5.E-04**	−0.21 ±0.04	**1.0.E-06**	−0.04 ±0.06	0.556	−0.06 ±0.06	0.245	−0.05 ±0.04	0.210
rs1785509*	G	intron	1.53 (1.17−2.01)	**2.2.E-03**	1.63 (1.13−2.37)	**0.010**	1.58 (1.27−1.97)	**4.5.E-05**	−0.21 ±0.06	**2.9.E-04**	−0.20 ±0.06	**5.7.E-04**	−0.21 ±0.04	**6.2.E-07**	−0.04 ±0.06	0.563	−0.08 ±0.05	0.139	−0.06 ±0.04	0.142
rs4521843*	T	intron	1.89 (1.24−2.87)	**2.9.E-03**	2.27 (1.28−4.02)	**4.9.E-03**	2.01 (1.44−2.82)	**4.4.E-05**	−0.28 ±0.09	**2.8.E-03**	−0.08 ±0.09	0.372	−0.18 ±0.07	**5.4.E-03**	0.00 ±0.10	0.978	−0.05 ±0.09	0.595	−0.02 ±0.06	0.752
rs1760816	A	intron	1.50 (1.14−1.99)	**4.3.E-03**	1.56 (1.07−2.27)	**0.022**	1.54 (1.23−1.92)	**1.9.E-04**	−0.21 ±0.06	**4.5.E-04**	−0.20 ±0.06	**8.0.E-04**	−0.20 ±0.04	**1.3.E-06**	−0.04 ±0.06	0.497	−0.06 ±0.06	0.246	−0.05 ±0.04	0.187
rs1629188	G	intron	1.50 (1.14−1.99)	**4.3.E-03**	1.56 (1.07−2.27)	**0.022**	1.54 (1.23−1.92)	**1.9.E-04**	−0.21 ±0.06	**4.5.E-04**	−0.20 ±0.06	**8.0.E-04**	−0.20 ±0.04	**1.3.E-06**	−0.04 ±0.06	0.497	−0.06 ±0.06	0.246	−0.05 ±0.04	0.187
rs1785507	C	intron	1.50 (1.14−1.99)	**4.3.E-03**	1.56 (1.07−2.27)	**0.022**	1.54 (1.23−1.92)	**1.9.E-04**	−0.21 ±0.06	**4.5.E-04**	−0.20 ±0.06	**8.0.E-04**	−0.20 ±0.04	**1.3.E-06**	−0.04 ±0.06	0.497	−0.06 ±0.06	0.246	−0.05 ±0.04	0.187
rs10814062	T	intron	1.50 (1.14−1.99)	**4.3.E-03**	1.56 (1.07−2.27)	**0.022**	1.54 (1.23−1.92)	**1.9.E-04**	−0.21 ±0.06	**4.5.E-04**	−0.20 ±0.06	**8.0.E-04**	−0.20 ±0.04	**1.3.E-06**	−0.04 ±0.06	0.497	−0.06 ±0.06	0.246	−0.05 ±0.04	0.187
rs1543605*	C	intron	1.51 (1.14−2.00)	**3.8.E-03**	1.57 (1.07−2.30)	**0.020**	1.55 (1.23−1.94)	**1.5.E-04**	−0.21 ±0.06	**4.0.E-04**	−0.20 ±0.06	**7.7.E-04**	−0.21 ±0.04	**1.1.E-06**	−0.04 ±0.06	0.508	−0.07 ±0.06	0.232	−0.06 ±0.04	0.185
rs1543604*	C	intron	1.46 (1.17−1.82)	**7.7.E-04**	1.39 (1.02−1.91)	**0.040**	1.45 (1.21−1.74)	**5.2.E-05**	−0.16 ±0.05	**5.6.E-04**	−0.11 ±0.05	**0.025**	−0.13 ±0.03	**5.7.E-05**	−0.06 ±0.05	0.186	−0.02 ±0.04	0.620	−0.04 ±0.03	0.177
rs747091	T	intron	1.50 (1.14−1.99)	**4.3.E-03**	1.56 (1.07−2.27)	**0.022**	1.54 (1.23−1.92)	**1.9.E-04**	−0.21 ±0.06	**4.5.E-04**	−0.20 ±0.06	**8.0.E-04**	−0.20 ±0.04	**1.3.E-06**	−0.04 ±0.06	0.497	−0.06 ±0.06	0.246	−0.05 ±0.04	0.187
rs10738921	G	intron	1.20 (0.85−1.70)	0.294	1.06 (0.65−1.74)	0.815	1.17 (0.88−1.55)	0.285	−0.15 ±0.07	**0.045**	−0.23 ±0.08	**2.3.E-03**	−0.19 ±0.05	**4.5.E-04**	−0.06 ±0.08	0.412	−0.04 ±0.07	0.550	−0.06 ±0.05	0.270
rs10117410	G	intron	1.50 (1.14−1.99)	**4.3.E-03**	1.56 (1.07−2.27)	**0.022**	1.54 (1.23−1.92)	**1.9.E-04**	−0.21 ±0.06	**4.5.E-04**	−0.20 ±0.06	**8.0.E-04**	−0.20 ±0.04	**1.3.E-06**	−0.04 ±0.06	0.497	−0.06 ±0.06	0.246	−0.05 ±0.04	0.187
rs10121647	T	intron	1.21 (0.86−1.71)	0.279	1.08 (0.66−1.77)	0.767	1.18 (0.89−1.56)	0.260	−0.15 ±0.07	**0.036**	−0.22 ±0.08	**3.1.E-03**	−0.19 ±0.05	**4.4.E-04**	−0.06 ±0.08	0.395	−0.04 ±0.07	0.621	−0.05 ±0.05	0.292
rs12342186	C	intron	1.18 (0.91−1.53)	0.204	1.05 (0.72−1.54)	0.794	1.15 (0.93−1.42)	0.206	−0.04 ±0.06	0.448	0.03 ±0.06	0.573	−0.01 ±0.04	0.865	−0.06 ±0.06	0.327	0.04 ±0.05	0.457	−0.01 ±0.04	0.787
rs10122495	T	upstream	1.51 (1.14−2.00)	**4.3.E-03**	1.58 (1.08−2.31)	**0.018**	1.54 (1.23−1.93)	**1.7.E-04**	−0.21 ±0.06	**3.9.E-04**	−0.20 ±0.06	**7.1.E-04**	−0.21 ±0.04	**1.0.E-06**	−0.04 ±0.06	0.540	−0.07 ±0.06	0.197	−0.06 ±0.04	0.175
rs10453201	C	upstream	1.43 (1.14−1.78)	**1.7.E-03**	1.39 (1.01−1.90)	**0.042**	1.43 (1.19−1.71)	**1.2.E-04**	−0.15 ±0.05	**8.6.E-04**	−0.11 ±0.05	**0.021**	−0.13 ±0.03	**6.6.E-05**	−0.06 ±0.05	0.204	−0.02 ±0.04	0.626	−0.04 ±0.03	0.192
rs10814064*	A	upstream	1.55 (1.17−2.04)	**2.3.E-03**	1.59 (1.09−2.32)	**0.017**	1.57 (1.25−1.97)	**8.3.E-05**	−0.22 ±0.06	**2.4.E-04**	−0.21 ±0.06	**4.4.E-04**	−0.21 ±0.04	**4.0.E-07**	−0.04 ±0.06	0.499	−0.08 ±0.06	0.177	−0.06 ±0.04	0.150
rs10814065	G	upstream	1.18 (0.92−1.52)	0.203	1.02 (0.70−1.48)	0.926	1.14 (0.92−1.40)	0.236	0.00 ±0.05	0.950	0.06 ±0.06	0.327	0.03 ±0.04	0.523	−0.08 ±0.06	0.143	0.01 ±0.05	0.899	−0.04 ±0.04	0.290

The *p*-values below the standard significance level ($p < 0.05$) are bolded. Abbreviations: A1, minor allele; Add, additive genetic model; MT-SOS T-score, bone density estimated by speed of sound (SOS) T-score at midshaft tibia; DR-SOS T-score, bone density estimated by SOS T-score at distal radius; MAF, minor allele frequency; *p*, *p*-value; se, standard error; and UTR, untranslated region. Source data are provided as a Source Data file.

Supplementary Table 4. Basic characteristics of the postmenopausal women subjects participating in the study and from which bone marrow samples were collected.

Characteristics	Controls	Osteoporosis	*p*-value*
no.	15	30	
Age (year)	66.00 ± 5.32	72.48 ± 5.44	0.007
Lumbar spine T score	−0.16 ± 0.53	−2.61 ± 1.28	< 0.0001
Femur neck T score	−0.05 ± 1.09	−2.53 ± 0.87	< 0.0001
Femur total T score	−0.04 ± 0.30	−2.41 ± 0.74	< 0.0001

*Significant differences in characteristics between two groups were determined by two-tailed Student's *t*-test.

Supplementary Table 5. Basic characteristics of the postmenopausal women subjects participating in the study and from which peripheral blood samples were collected.

Characteristics	Controls	Osteoporosis	*p*- value*
no.	32	31	
Age (year)	54.31 ± 4.25	56.81 ± 4.50	0.026
Lumbar spine T score	0.27 ± 0.72	−2.77 ± 0.55	< 0.0001
Femur neck T score	−0.13 ± 0.63	−2.01 ± 0.70	< 0.0001
Total hip T score	0.32 ± 0.81	−1.79 ± 0.70	< 0.0001

*Significant differences in characteristics between two groups were determined by two-tailed Student's *t*-test.

Supplementary Table 6. Results of association analysis of the 96 SNPs (17 genotyped and 79 imputed* SNPs) in the *UBAP2* region associated with osteoporosis, bone density, and obesity in the Korean women subjects.

SNP	A1	Quantitative analysis for MT-SOS T-score (n=3,569; covariates: area, age, BMI)		Osteoporosis case-control analysis (controls=2,223:cases=443) (covariates: area, age, BMI)		Quantitative analysis for BMI (n=3,803; covariates: area, age)		Obesity case-control analysis (controls=2,163:cases=1,611) (covariates: area, age)	
		beta ± se	Add p	OR (95% CI)	Add p	beta ± se	Add p	OR (95% CI)	Add p
rs7022524	T	-0.18 ±0.05	**5.81E-04**	1.14 (1.52-0.89)	0.371	0.14 ±0.12	0.259	1.08 (0.93-1.26)	0.286
rs4817	C	-0.19 ±0.04	**4.08E-06**	1.51 (1.90-3.53)	**4.13E-04**	0.16 ±0.10	0.099	1.14 (1.01-1.28)	**3.45E-02**
rs16935278	C	-0.18 ±0.05	**6.23E-04**	1.14 (1.52-0.90)	0.369	0.13 ±0.12	0.284	1.08 (0.93-1.26)	0.290
rs16935280	C	-0.18 ±0.05	**6.23E-04**	1.14 (1.52-0.90)	0.369	0.13 ±0.12	0.284	1.08 (0.93-1.26)	0.290
rs11848*	C	-0.05 ±0.10	0.567	1.08 (1.76-0.29)	0.770	0.09 ±0.23	0.686	0.91 (0.68-1.21)	0.507
rs2277178*	A	-0.11 ±0.06	0.070	1.10 (1.54-0.54)	0.587	0.10 ±0.14	0.495	1.06 (0.89-1.26)	0.529
rs2781*	C	-0.20 ±0.04	**4.42E-07**	1.68 (2.08-4.71)	**2.43E-06**	0.16 ±0.09	0.086	1.14 (1.02-1.28)	**2.48E-02**
rs2296365	C	-0.18 ±0.05	**6.14E-04**	1.15 (1.53-0.92)	0.355	0.12 ±0.12	0.310	1.08 (0.93-1.25)	0.310
rs307695	G	-0.18 ±0.05	**5.92E-04**	1.15 (1.53-0.92)	0.355	0.12 ±0.12	0.310	1.08 (0.93-1.25)	0.309
rs307694	A	-0.20 ±0.04	**8.76E-07**	1.61 (2.01-4.18)	**2.93E-05**	0.13 ±0.10	0.176	1.13 (1.00-1.27)	**4.98E-02**
rs10971796	A	-0.16 ±0.06	**4.83E-03**	2.03 (2.75-4.52)	**6.10E-06**	0.10 ±0.14	0.476	1.14 (0.96-1.34)	0.136
rs307693	A	-0.18 ±0.05	**5.92E-04**	1.15 (1.53-0.92)	0.355	0.12 ±0.12	0.310	1.08 (0.93-1.25)	0.309
rs745532	A	-0.20 ±0.04	**8.76E-07**	1.61 (2.01-4.18)	**2.93E-05**	0.13 ±0.10	0.176	1.13 (1.00-1.27)	**4.98E-02**
rs307691	C	-0.20 ±0.04	**1.02E-06**	1.61 (2.00-4.17)	**3.00E-05**	0.13 ±0.10	0.186	1.12 (1.00-1.26)	0.053
rs307690	T	-0.18 ±0.05	**6.04E-04**	1.15 (1.53-0.92)	0.355	0.12 ±0.12	0.315	1.08 (0.93-1.25)	0.314
rs307689	A	-0.18 ±0.05	**6.04E-04**	1.15 (1.53-0.92)	0.355	0.12 ±0.12	0.315	1.08 (0.93-1.25)	0.314
rs10120819	C	-0.16 ±0.06	**4.93E-03**	2.03 (2.75-4.53)	**6.04E-06**	0.10 ±0.14	0.479	1.14 (0.96-1.34)	0.135
rs307688	G	-0.20 ±0.04	**1.06E-06**	1.61 (2.00-4.17)	**3.00E-05**	0.12 ±0.10	0.189	1.12 (1.00-1.26)	0.054
rs307687*	C	-0.15 ±0.05	**3.57E-03**	1.16 (1.53-1.03)	0.302	0.10 ±0.12	0.403	1.04 (0.90-1.20)	0.604
rs307642	T	-0.20 ±0.04	**1.06E-06**	1.61 (2.00-4.17)	**3.00E-05**	0.12 ±0.10	0.189	1.12 (1.00-1.26)	0.054
rs307645	A	-0.20 ±0.04	**1.06E-06**	1.61 (2.00-4.17)	**3.00E-05**	0.12 ±0.10	0.189	1.12 (1.00-1.26)	0.054
rs307646*	G	-0.20 ±0.04	**8.95E-07**	1.63 (2.03-4.35)	**1.35E-05**	0.11 ±0.09	0.237	1.11 (0.99-1.24)	0.078
rs307647	G	-0.20 ±0.04	**1.06E-06**	1.61 (2.00-4.17)	**3.00E-05**	0.12 ±0.10	0.189	1.12 (1.00-1.26)	0.054
rs307648	C	-0.20 ±0.04	**1.06E-06**	1.61 (2.00-4.17)	**3.00E-05**	0.12 ±0.10	0.189	1.12 (1.00-1.26)	0.054
rs307649	T	-0.19 ±0.04	**4.45E-06**	1.52 (1.91-3.55)	**3.83E-04**	0.15 ±0.10	0.114	1.14 (1.01-1.29)	**3.14E-02**
rs874572*	G	-0.16 ±0.06	**7.49E-03**	1.95 (2.69-4.08)	**4.52E-05**	0.16 ±0.14	0.263	1.19 (1.00-1.41)	0.055
rs307650	A	-0.18 ±0.05	**6.04E-04**	1.15 (1.53-0.92)	0.355	0.12 ±0.12	0.315	1.08 (0.93-1.25)	0.314
rs12377078*	A	-0.17 ±0.19	0.367	2.08 (4.76-1.73)	0.083	0.86 ±0.45	0.057	1.63 (0.94-2.85)	0.085
rs307651	A	-0.19 ±0.04	**4.89E-06**	1.50 (1.89-3.49)	**4.86E-04**	0.15 ±0.10	0.118	1.14 (1.01-1.28)	**3.63E-02**
rs307652*	A	0.04 ±0.04	0.344	1.07 (1.32-0.64)	0.522	0.02 ±0.09	0.838	0.95 (0.85-1.06)	0.316
rs307657	C	-0.19 ±0.04	**4.89E-06**	1.50 (1.89-3.49)	**4.86E-04**	0.15 ±0.10	0.118	1.14 (1.01-1.28)	**3.63E-02**
rs307658	C	-0.19 ±0.04	**4.89E-06**	1.50 (1.89-3.49)	**4.86E-04**	0.15 ±0.10	0.118	1.14 (1.01-1.28)	**3.63E-02**
rs307660	C	0.03 ±0.04	0.424	1.10 (1.35-0.85)	0.395	0.03 ±0.09	0.751	0.95 (0.85-1.06)	0.331
rs307654	T	0.00 ±0.04	0.932	1.11 (1.39-0.96)	0.337	0.03 ±0.09	0.714	0.95 (0.84-1.06)	0.360
rs595556	C	-0.19 ±0.04	**4.89E-06**	1.50 (1.89-3.49)	**4.86E-04**	0.15 ±0.10	0.118	1.14 (1.01-1.28)	**3.63E-02**
rs10971818*	A	-0.21 ±0.13	0.120	2.07 (3.64-2.55)	**1.09E-02**	0.82 ±0.31	**9.15E-03**	1.68 (1.14-2.49)	**9.40E-03**
rs16935299	T	-0.12 ±0.03	**3.94E-04**	1.40 (1.69-3.56)	**3.65E-04**	0.12 ±0.08	0.123	1.05 (0.95-1.15)	0.342
rs558415	C	-0.18 ±0.05	**6.04E-04**	1.15 (1.53-0.92)	0.355	0.12 ±0.12	0.315	1.08 (0.93-1.25)	0.314
rs544169	G	-0.19 ±0.04	**3.96E-06**	1.51 (1.90-3.54)	**4.04E-04**	0.16 ±0.10	0.107	1.14 (1.01-1.28)	**3.24E-02**
rs668071	C	-0.19 ±0.04	**3.96E-06**	1.51 (1.90-3.54)	**4.04E-04**	0.16 ±0.10	0.107	1.14 (1.01-1.28)	**3.24E-02**
rs10971822	A	-0.17 ±0.06	**5.85E-03**	1.95 (2.69-4.08)	**4.49E-05**	0.17 ±0.14	0.240	1.19 (1.00-1.42)	0.051
rs307661	T	0.00 ±0.04	0.935	1.16 (1.44-1.33)	0.183	0.08 ±0.09	0.397	0.97 (0.87-1.09)	0.644
rs307663	G	-0.18 ±0.05	**6.91E-04**	1.14 (1.52-0.90)	0.368	0.12 ±0.12	0.312	1.08 (0.93-1.25)	0.312
rs307665	G	-0.18 ±0.05	**6.91E-04**	1.14 (1.52-0.90)	0.368	0.12 ±0.12	0.312	1.08 (0.93-1.25)	0.312
rs307670	T	-0.19 ±0.04	**3.96E-06**	1.51 (1.90-3.54)	**4.04E-04**	0.16 ±0.10	0.107	1.14 (1.01-1.28)	**3.24E-02**
rs307678	A	-0.19 ±0.04	**3.96E-06**	1.51 (1.90-3.54)	**4.04E-04**	0.16 ±0.10	0.107	1.14 (1.01-1.28)	**3.24E-02**
rs307679	C	-0.18 ±0.05	**6.91E-04**	1.14 (1.52-0.90)	0.368	0.12 ±0.12	0.312	1.08 (0.93-1.25)	0.312
rs307680	C	-0.18 ±0.05	**6.91E-04**	1.14 (1.52-0.90)	0.368	0.12 ±0.12	0.312	1.08 (0.93-1.25)	0.312
rs3739690	A	-0.12 ±0.03	**2.21E-04**	1.43 (1.72-3.80)	**1.43E-04**	0.15 ±0.08	0.051	1.06 (0.97-1.17)	0.197
rs307681	A	-0.18 ±0.05	**6.91E-04**	1.14 (1.52-0.90)	0.368	0.12 ±0.12	0.312	1.08 (0.93-1.25)	0.312
rs307682*	G	-0.19 ±0.04	**6.78E-06**	1.50 (1.89-3.48)	**5.09E-04**	0.14 ±0.10	0.137	1.14 (1.01-1.28)	**3.56E-02**
rs307683	C	-0.19 ±0.04	**3.96E-06**	1.51 (1.90-3.54)	**4.04E-04**	0.16 ±0.10	0.107	1.14 (1.01-1.28)	**3.24E-02**
rs4272470	G	-0.19 ±0.04	**3.96E-06**	1.51 (1.90-3.54)	**4.04E-04**	0.16 ±0.10	0.107	1.14 (1.01-1.28)	**3.24E-02**
rs307697	C	-0.19 ±0.04	**3.96E-06**	1.51 (1.90-3.54)	**4.04E-04**	0.16 ±0.10	0.107	1.14 (1.01-1.28)	**3.24E-02**
rs307699*	T	-0.17 ±0.05	**8.98E-04**	1.13 (1.51-0.84)	0.400	0.11 ±0.12	0.339	1.07 (0.93-1.25)	0.347
rs307700	G	-0.19 ±0.04	**3.96E-06**	1.51 (1.90-3.54)	**4.04E-04**	0.16 ±0.10	0.107	1.14 (1.01-1.28)	**3.24E-02**
rs307703	G	0.02 ±0.04	0.542	1.15 (1.41-1.28)	0.202	0.07 ±0.09	0.464	0.97 (0.87-1.09)	0.615
rs834027	T	-0.19 ±0.04	**3.96E-06**	1.51 (1.90-3.54)	**4.04E-04**	0.15 ±0.10	0.109	1.14 (1.01-1.28)	**3.32E-02**
rs10971836	C	-0.12 ±0.03	**2.24E-04**	1.43 (1.72-3.81)	**1.39E-04**	0.15 ±0.08	**4.98E-02**	1.07 (0.97-1.17)	0.170
rs6476429	A	-0.19 ±0.04	**3.96E-06**	1.51 (1.90-3.54)	**4.04E-04**	0.15 ±0.10	0.109	1.14 (1.01-1.28)	**3.32E-02**
rs10814058	G	-0.19 ±0.04	**3.96E-06**	1.51 (1.90-3.54)	**4.04E-04**	0.15 ±0.10	0.109	1.14 (1.01-1.28)	**3.32E-02**
rs932969	C	-0.17 ±0.06	**5.85E-03**	1.95 (2.69-4.08)	**4.49E-05**	0.17 ±0.14	0.242	1.19 (1.00-1.42)	0.051
rs7019441	A	-0.18 ±0.05	**3.92E-04**	1.22 (1.60-1.42)	0.155	0.18 ±0.12	0.128	1.11 (0.96-1.29)	0.142
rs10814059	T	-0.19 ±0.04	**3.96E-06**	1.51 (1.90-3.54)	**4.04E-04**	0.15 ±0.10	0.109	1.14 (1.01-1.28)	**3.32E-02**
rs10814060	G	-0.19 ±0.04	**3.96E-06**	1.51 (1.90-3.54)	**4.04E-04**	0.15 ±0.10	0.109	1.14 (1.01-1.28)	**3.32E-02**
rs12555291	A	0.00 ±0.04	0.935	1.16 (1.44-1.33)	0.183	0.08 ±0.09	0.397	0.97 (0.87-1.09)	0.644
rs7029112	C	-0.19 ±0.04	**3.96E-06**	1.51 (1.90-3.54)	**4.04E-04**	0.15 ±0.10	0.109	1.14 (1.01-1.28)	**3.32E-02**
rs7029122	C	-0.18 ±0.05	**6.91E-04**	1.14 (1.52-0.90)	0.368	0.12 ±0.12	0.315	1.08 (0.93-1.25)	0.316
rs10465080	T	-0.10 ±0.03	**1.72E-03**	1.42 (1.71-3.73)	**1.95E-04**	0.13 ±0.08	0.082	1.05 (0.96-1.15)	0.305
rs10758236	C	-0.19 ±0.04	**3.96E-06**	1.51 (1.90-3.54)	**4.04E-04**	0.15 ±0.10	0.109	1.14 (1.01-1.28)	**3.32E-02**
rs3016756	C	-0.12 ±0.03	**2.21E-04**	1.43 (1.72-3.80)	**1.43E-04**	0.15 ±0.08	0.053	1.06 (0.97-1.17)	0.202
rs1785506	C	-0.19 ±0.04	**3.96E-06**	1.51 (1.90-3.54)	**4.04E-04**	0.15 ±0.10	0.109	1.14 (1.01-1.28)	**3.32E-02**
rs1785503	C	-0.19 ±0.04	**3.96E-06**	1.51 (1.90-3.54)	**4.04E-04**	0.15 ±0.10	0.109	1.14 (1.01-1.28)	**3.32E-02**
rs12553933	G	0.00 ±0.04	0.935	1.16 (1.44-1.33)	0.183	0.08 ±0.09	0.397	0.97 (0.87-1.09)	0.644
rs12164256	G	-0.10 ±0.03	**1.72E-03**	1.42 (1.71-3.73)	**1.95E-04**	0.13 ±0.08	0.083	1.05 (0.96-1.15)	0.307
rs1785502	C	-0.19 ±0.04	**3.96E-06**	1.51 (1.90-3.54)	**4.04E-04**	0.15 ±0.10	0.111	1.14 (1.01-1.28)	**3.46E-02**
rs1785500	C	-0.19 ±0.04	**3.96E-06**	1.51 (1.90-3.54)	**4.04E-04**	0.15 ±0.10	0.111	1.14 (1.01-1.28)	**3.46E-02**
rs1758632	C	-0.19 ±0.04	**3.96E-06**	1.51 (1.90-3.54)	**4.04E-04**	0.15 ±0.10	0.111	1.14 (1.01-1.28)	**3.46E-02**
rs1785512	C	-0.19 ±0.04	**3.96E-06**	1.51 (1.90-3.54)	**4.04E-04**	0.15 ±0.10	0.111	1.14 (1.01-1.28)	**3.46E-02**
rs1758633*	C	-0.19 ±0.04	**3.18E-06**	1.51 (1.90-3.57)	**3.52E-04**	0.15 ±0.10	0.114	1.14 (1.01-1.28)	**3.55E-02**
rs1785509*	G	-0.19 ±0.04	**2.49E-06**	1.55 (1.94-3.88)	**1.05E-04**	0.18 ±0.09	0.055	1.16 (1.03-1.30)	**1.58E-02**
rs4521843*	T	-0.17 ±0.06	**8.25E-03**	2.01 (2.83-3.99)	**6.50E-05**	0.13 ±0.15	0.393	1.20 (1.00-1.45)	0.052
rs1760816	A	-0.19 ±0.04	**3.96E-06**	1.51 (1.90-3.54)	**4.04E-04**	0.15 ±0.10	0.111	1.14 (1.01-1.28)	**3.46E-02**
rs1629188	G	-0.19 ±0.04	**3.96E-06**	1.51 (1.90-3.54)	**4.04E-04**	0.15 ±0.10	0.111	1.14 (1.01-1.28)	**3.46E-02**
rs1785507	C	-0.19 ±0.04	**3.96E-06**	1.51 (1.90-3.54)	**4.04E-04**	0.15 ±0.10	0.111	1.14 (1.01-1.28)	**3.46E-02**
rs10814062	T	-0.19 ±0.04	**3.96E-06**	1.51 (1.90-3.54)	**4.04E-04**	0.15 ±0.10	0.111	1.14 (1.01-1.28)	**3.46E-02**
rs1543605*	C	-0.19 ±0.04	**3.22E-06**	1.52 (1.91-3.59)	**3.26E-04**	0.15 ±0.10	0.126	1.13 (1.00-1.27)	0.051
rs1543604*	C	-0.12 ±0.03	**1.70E-04**	1.45 (1.75-3.96)	**7.57E-05**	0.15 ±0.08	0.050	1.06 (0.97-1.17)	0.220
rs747091	T	-0.20 ±0.04	**1.91E-06**	1.53 (1.92-3.62)	**3.01E-04**	0.15 ±0.10	0.127	1.13 (1.00-1.27)	0.055
rs10738921	G	-0.18 ±0.05	**6.91E-04**	1.14 (1.52-0.90)	0.368	0.12 ±0.12	0.315	1.08 (0.93-1.25)	0.316
rs10117410	G	-0.19 ±0.04	**3.96E-06**	1.51 (1.90-3.54)	**4.04E-04**	0.15 ±0.10	0.111	1.14 (1.01-1.28)	**3.46E-02**
rs10121647	T	-0.18 ±0.05	**7.58E-04**	1.15 (1.53-0.93)	0.353	0.14 ±0.12	0.235	1.09 (0.94-1.27)	0.250
rs12342186	C	0.00 ±0.04	0.953	1.15 (1.43-1.30)	0.194	0.08 ±0.09	0.388	0.97 (0.87-1.09)	0.654
rs10122495	T	-0.19 ±0.04	**3.26E-06**	1.52 (1.91-3.58)	**3.48E-04**	0.16 ±0.10	0.099	1.14 (1.01-1.28)	**3.26E-02**
rs10453201	C	-0.12 ±0.03	**1.95E-04**	1.42 (1.71-3.76)	**1.72E-04**	0.15 ±0.08	**4.86E-02**	1.06 (0.97-1.17)	0.197
rs10814064*	A	-0.20 ±0.04	**1.40E-06**	1.54 (1.94-3.73)	**1.92E-04**	0.17 ±0.10	0.080	1.15 (1.02-1.29)	**2.35E-02**
rs10814065	G	0.03 ±0.04	0.478	1.14 (1.41-1.19)	0.234	0.05 ±0.09	0.570	0.96 (0.86-1.07)	0.480

The *p*-values below the standard significance level ($p < 0.05$) are bolded and underlined. Abbreviations: A1, minor allele; Add, additive genetic model; BMI, body mass index; MT-SOS T-score, bone density estimated by speed of sound (SOS) T-score at midshaft tibia; OR, odds ration; *p*, *p*-value; se, standard error. Source data are provided as a Source Data file.

Supplementary Table 7. Target sequences of shRNAs used to knockdown gene expression.

Gene symbol	Name	Target sequence (5′ to 3′)
Ubap2	sh*Ubap2* _#1	ACGCTTACAGCACAGGTTATG
	sh*Ubap2* _#2	GATGGGAATCTAGCTAATAAT
Cdh1	sh*Cdh1* _#1	GCTGGAATCTTTGTCCATGTA
	sh*Cdh1* _#2	CGGGACAATGTGTATTACTAT
	sh*Cdh1* _#3	GCCTCATATCATCACCATCTT
	sh*Cdh1* _#4	CCGAGAGAGTTACCCTACATA
	sh*Cdh1* _#5	CCACGACCAATGATGGCATTT
Fosl1	sh*Fosl1* _#1	GCTCTCCTACACTCCTGGCTT
	sh*Fosl1* _#2	CCAGGAGTCATACGAGCCCTA
	sh*Fosl1* _#3	CCAGTGCCTTGCATCTCCCTT
	sh*Fosl1* _#4	AGCAGCAGAAGTTCCACCTTG
	sh*Fosl1* _#5	CGACAAATTGGAGGATGAGAA

Supplementary Table 8. List of primers used for quantitative reverse-transcriptase polymerase chain reaction.

Species	Gene name	GenBank No.	Forward primers (5′ to 3′)	Reverse primers (5′ to 3′)
Mus musculus	*Ubap2*	NM_026872	TGAGCAATGATCGTTGCCGAG	ACTTGAGCAAGCCGCATCT
	Alpl	NM_007431	CCAACTCTTTTGTGCCAGAGA	TGACATTCTTGGCTACATTGGTG
	Runx2	NM_009820	TAAAGTGACAGTGGACGGTCCC	TGCGCCCTAAATCACTGAGG
	Bglap	NM_007541	TAGTGAACAGACTCCGGCGCT	TGTAGGCGGTCTTCAAGCCAT
	Sp7	NM_130458	ATGGCGTCCTCTCTGCTTG	TGAAAGGTCAGCGTATGGCTT
	Acp5	NM_001102405	TGGTATGTGCTGGCTGGAAAC	AGTTGCCACACAGCATCACTG
	Ctsk	NM_007802	GAAGAAGACTCACCAGAAGCA	TCCAGGTTATGGGCAGAGATT
	Gusb	NM_001289726	TGACCACAGTCCATGCCATC	GACGGACACATTGGGGGTAG
Homo sapiens	*UBAP2*	NM_018449	AGTGGCCCTACATGATTCTAA	TCTCCCATGAAGTTGTGTCTG
	ALPL	NM_000478	GGGACTGGTACTCAGACAACG	GTAGGCGATGTCCTTACAGCC
	BGLAP	NM_199173	GTGCAGAGTCCAGCAAAGGTG	CAACTCGTCACAGTCCGGATTG
	TNF	NM_000594	CCTCTCTCTAATCAGCCCTCTG	GAGGACCTGGGAGTAGATGAG
	ACP5	NM_0011103	TGAGGACGTATTCTCTGACCG	CACATTGGTCTGTGGGATCTTG
	CTSK	NM_000396	GCAGAAGAACCGGGGTATTGACT	GGAAGGAGGTCAGGCTTGCATC
	GUSB	NM_000181	TCCGTATGTGGATGTGATCTGT	ATCAGAGGTGGATCCTGGTG

Supplementary References

1. Cho YS, *et al.* A large-scale genome-wide association study of Asian populations uncovers genetic factors influencing eight quantitative traits. *Nat Genet* **41**, 527-534 (2009).

2. Robin X, *et al.* pROC: an open-source package for R and S+ to analyze and compare ROC curves. *BMC Bioinform* **12**, 77 (2011).
