# Supplementary material for: UBAP2 plays a role in bone homeostasis through the regulation of osteoblastogenesis and osteoclastogenesis
Source: Nat Commun. 2023 Jun 20;14:3668. doi: 10.1038/s41467-023-39448-8 (PMC10281941; doi:10.1038/s41467-023-39448-8)
Supplement: Supplementary file 3 — Reporting Summary [file 41467_2023_39448_MOESM3_ESM.pdf]

nature portfolio

Corresponding author(s): Seon-Yong Jeong, Yoon-Sok Chung, Hyun-Seok Jin

Last updated by author(s): Jun 5, 2023

Reporting Summary

Nature Portfolio wishes to improve the reproducibility of the work that we publish. This form provides structure for consistency and transparency in reporting. For further information on Nature Portfolio policies, see our Editorial Policies and the Editorial Policy Checklist.

Statistics

For all statistical analyses, confirm that the following items are present in the figure legend, table legend, main text, or Methods section.

n/a	Confirmed	
☐	☒	The exact sample size (*n*) for each experimental group/condition, given as a discrete number and unit of measurement
☐	☒	A statement on whether measurements were taken from distinct samples or whether the same sample was measured repeatedly
☐	☒	The statistical test(s) used AND whether they are one- or two-sided<br>*Only common tests should be described solely by name; describe more complex techniques in the Methods section.*
☐	☒	A description of all covariates tested
☐	☒	A description of any assumptions or corrections, such as tests of normality and adjustment for multiple comparisons
☐	☒	A full description of the statistical parameters including central tendency (e.g. means) or other basic estimates (e.g. regression coefficient) AND variation (e.g. standard deviation) or associated estimates of uncertainty (e.g. confidence intervals)
☐	☒	For null hypothesis testing, the test statistic (e.g. *F*, *t*, *r*) with confidence intervals, effect sizes, degrees of freedom and *P* value noted<br>*Give P values as exact values whenever suitable.*
☒	☐	For Bayesian analysis, information on the choice of priors and Markov chain Monte Carlo settings
☒	☐	For hierarchical and complex designs, identification of the appropriate level for tests and full reporting of outcomes
☒	☐	Estimates of effect sizes (e.g. Cohen's *d*, Pearson's *r*), indicating how they were calculated

Our web collection on statistics for biologists contains articles on many of the points above.

Software and code

Policy information about availability of computer code

Data collection

The statistical analysis of the association study results was performed using the whole-genome association analysis toolset PLINK version 1.07 (https://zzz.bwh.harvard.edu/plink/) and PASW Statistics software version 17.0 (SPSS Inc., Chicago, IL, USA).
LocusZoom version 0.5.3 (http://locuszoom.sph.umich.edu/) and the SNP Annotation and Proxy search (SNAP) database (http://www.broadinstitute.org/mpg/snap/) were used for regional association plot construction.
The Affymetrix Genome-Wide Human SNP array 5.0 (Affymetrix, Santa Clara, CA, USA) was used for SNP genotyping. Seventy-nine imputed SNPs in UBAP2 were selected using genotype imputation analysis with MACH 1.0.16.
Luminescence was measured using a luminometer (Turner Design Instrument, Sunnyvale, CA, USA).
Isolated monocytes from mouse bone marrow were confirmed using the FACS Aria III cell sorter (BD Biosciences, San Jose, CA, USA) and FACS Diva software version 8.0.3 (BD Biosciences).
The absorbance of samples in the ALP/TRAP assay was read using the iMark™ Microplate Absorbance Reader (Microplate Manager 6 software; Bio-Rad).
The analysis of qRT-PCR was performed using QIAGEN Rotor-Q software (version 2.0.2) or Bio-Rad CFX Manager™ software (Bio-Rad Laboratories).
For western blot analysis, iBrigntFL1000 (Invitrogen by Thermo Fisher Scientific) was used.
The protein band intensities for quantitative analysis were normalized to β-actin level using Image Processing and Analysis in Java (Image J) software (ImageJ bundled with 64-bit Java 8; http://imagej.nih.gov/ij/).
Images of zebrafish experiments were compiled using Affinity Desinger (version 1.10.6) and Affinity Photo (version 1.10.6).

Data analysis

A GWAS was performed with the additive genetic model, and P-values were adjusted for multiple tests using the Bonferroni-corrected significance level ($p < 7.7 \times 10$-6). A replication association analysis was performed with the additive genetic model, and results with $p < 0.05$ were considered statistically significant.

In the experimental studies, all experiments were repeated independently at least three times unless stated otherwise, and the results are presented as mean ± SD as indicated. Statistical analyses were performed using Prism version 9 (GraphPad) and statistical significance between the groups was calculated using Student's t-test, an unpaired two-tailed t-test, and a one-way analysis of variance (ANOVA) or two-way ANOVA for multiple comparisons. Results with $p < 0.05$ were considered statistically significant. Comparisons of multiple groups were performed using a one-way ANOVA, followed by Tukey's honest significant difference post-hoc test for correction of multiple comparison results.

For manuscripts utilizing custom algorithms or software that are central to the research but not yet described in published literature, software must be made available to editors and reviewers. We strongly encourage code deposition in a community repository (e.g. GitHub). See the Nature Portfolio guidelines for submitting code & software for further information.

Data

Policy information about availability of data

All manuscripts must include a data availability statement. This statement should provide the following information, where applicable:

- Accession codes, unique identifiers, or web links for publicly available datasets
- A description of any restrictions on data availability
- For clinical datasets or third party data, please ensure that the statement adheres to our policy

The exome-wide association study was performed using the dataset (subject information and SNP genotype data) of 2,666 women, which was originally generated in the KARE project (total 8,840 subjects) of KoGES [https://www.nih.go.kr/ko/main/contents.do?menuNo=300563] supported by the Korean National Institute of Health (KNIH), which is an affiliated organization of the Korea Disease Control and Prevention Agency (KDCA). The subject information and SNP genotype data used in this study are owned entirely by the KNIH, and disclosure of the raw data to the public without permission is strictly prohibited. Although the personal raw data of SNP genotype and epidemiology and subject information cannot be publicly shared because of legal restriction imposed by the Korean Bioethics and Safety Act, these data (with anonymization) can be used for research purpose. In principle, the raw data of subject information and SNP genotype used in this study are available with permission from the Institutional Review Board of KNIH [https://www.nih.go.kr/eng/main/main.do] for researchers in Korea who meet confidential data access criteria. These data can also be available for researchers overseas when undertaking an international cooperative research project and when the KDCA approves it. To access more information for data distribution, please refer to the Korea Biobank Project [https://www.kdca.go.kr/contents.es?mid=a30326000000]. All data supporting the findings described in this manuscript are available in the article and in the Supplementary Information and from the corresponding author upon request. Source data are provided with this paper.
Ensemble gene ID: ENSG00000137073, ENSDARG00000088318, and ENSDARG00000060065

Human research participants

Policy information about studies involving human research participants and Sex and Gender in Research.

Reporting on sex and gender	As the prevalence of osteoporosis is markedly higher in women than in men [PMID: 22532494 and PMID: 34657598], sex was considered in the following study designs. For the human sample study, sex of the participants was determined based on self-report. The basic characteristics of the participants are shown in Supplementary Tables 1, 4, and 5.
Population characteristics	All participants of this study were women aged above 50 years. To collect bone marrow samples, 45 postmenopausal women were recruited. The average age of the 15 controls was 66 years, and that of the 30 patients with osteoporosis was 72 years. The BMD (g cm–2) at the lumbar spine (L1–L4), femur, and hip was measured using X-ray absorptiometry (GE Lunar, Madison, WI, USA). The T score of the 30 patients with osteoporosis in the lumbar spine and femur neck was -2.61 ± 1.28 and -2.53 ± 0.87, respectively, which were significantly different from the values of the 15 controls.<br>To collect peripheral blood samples, 63 postmenopausal women were recruited. The average age of the 32 controls was 54.31 years, and that of the 31 patients with osteoporosis was 56.81 years. The T score of the 31 patients with osteoporosis in the lumbar spine and femur neck was -2.77 ± 0.55 and -2.01 ± 0.70, respectively, which were significantly different from the values of the 32 controls.
Recruitment	We recruited women (45 women for bone marrow samples and 63 women for peripheral blood samples) who agreed to sample collection at the Departments of Endocrinology and Metabolism, Family Practice and Community Health, and Orthopedic Surgery in Ajou University Hospital.
Ethics oversight	This research complies with relevant ethical regulations. Human sample studies were approved by the Ajou University Hospital IRB (approval numbers: AJIRB-GEN-GEN-11-062 and AJIRB-GEN-GEN-11-332), and written informed consent was obtained from all subjects.

Note that full information on the approval of the study protocol must also be provided in the manuscript.

Field-specific reporting

Please select the one below that is the best fit for your research. If you are not sure, read the appropriate sections before making your selection.

☒ Life sciences ☐ Behavioural & social sciences ☐ Ecological, evolutionary & environmental sciences

For a reference copy of the document with all sections, see nature.com/documents/nr-reporting-summary-flat.pdf

Life sciences study design

All studies must disclose on these points even when the disclosure is negative.

Sample size	In the association study (Table 1), the Quanto program (version 1.2.4) was used to determine whether the sample number would be sufficient; we found that at least 269 cases were required to satisfy 80% sample power. Four hundred and forty-three cases were included in the association study; hence, the number of cases was sufficient. For in vitro studies, at least three independent samples were used in each experiment, and cell numbers were calculated at least twice based on similar published studies .<br>Zebrafish experiments were designed in a dose-dependent manner of ubap2-morpholino RNA. Biologically independent embryos (n = 17–21) per experimental condition were used, which generally yields a strong statistical power for similar experiments. Sample sizes were calculated using the size power analysis for a prior determination, on the basis of SD and effect size previously obtained using the experimental methods used in the study.
Data exclusions	No data exclusions
Replication	Replication analysis of the identified significant SNPs was performed in 1371 participants of another Korean woman cohort (Ansan) and all experiments were repeated two or three times with duplicated determinations.
Randomization	The groups for in vitro experiments with MC3T3-E1 cells and primary monocytes were designed as follows: Control (no induction), Mock (no manipulation of any genes), shCont (pLKO.1-puro empty vector), Vector (pDON-5 Neo vector), shUbap2_#1 (shRNA against Ubap2 showing over 80% knockdown (K/D) efficiency of Ubap2), shUbap2_#2 (shRNA against Ubap2 showing 40%–50% K/D efficiency of Ubap2), and Ubap2 (Ubap2 overexpression).<br>For experiments performed with human samples, the patient group with osteoporosis and the normal control group were compared, and the average age between the groups was similar.<br>For zebrafish experiments, there was no specific order of sample assignment (random allocation).
Blinding	The investigators were not blinded to the in vitro experiments and human sample collections because it was necessary to distinguish the control and patient groups. For zebrafish experiments, sample labeling was principally blinded using sample numbers, instead of using names. When blind labeling was not possible, all efforts were made to apply identical conditions (such as treatment time and exposure time) to samples. The investigators conducted the experiments with zebrafish and ELISA of human samples in a blind manner.

Reporting for specific materials, systems and methods

We require information from authors about some types of materials, experimental systems and methods used in many studies. Here, indicate whether each material, system or method listed is relevant to your study. If you are not sure if a list item applies to your research, read the appropriate section before selecting a response.

Materials & experimental systems

n/a	Involved in the study	
☐	☒	Antibodies
☐	☒	Eukaryotic cell lines
☒	☐	Palaeontology and archaeology
☐	☒	Animals and other organisms
☒	☐	Clinical data
☒	☐	Dual use research of concern

Methods

n/a	Involved in the study	
☒	☐	ChIP-seq
☐	☒	Flow cytometry
☒	☐	MRI-based neuroimaging

Antibodies

Antibodies used	Rabbit polyclonal anti-Ubap2 was used for western blotting and ICC/IF (1:500 for WB, 1:100 for ICC; Abcepta Inc. formerly ABGENT; AP12773a).<br>Rabbit polyclonal anti-Ubap2 was used for western blotting (1:1000; Abcam; ab197083; Lot. 1021951-1).<br>Rabbit polyclonal anti-Ubap2 was used for western blotting (1:1000; Bethyl Laboratories Inc; A304-626A; Lot. #1).<br>Rabbit polyclonal anti-E-cadherin1 was used for western blotting (1:1000; Proteintech; 20874-1-ap; Lot. 00100170).<br>Mouse monoclonal anti-Fra1 was used for western blotting (1:1000; Santa Cruz Biotechnology; sc-28310; Lot. #G2921).<br>Mouse monoclonal anti-β-actin was used for western blotting (1:2500; Santa Cruz Biotechnology; sc-47778; Lot #1620).<br>HRP-conjugated goat anti-rabbit IgG was used for western blotting (1:10000; BETHYL; A120-101P; Lot. #40). HRP-conjugated goat anti-mouse IgG was used for western blotting (1:10000; BETHYL; A90-116P; Lot. #44). Goat anti-rabbit IgG H&L (FITC) was used for ICC/IF (1:2000; Abcam; ab6717)<br>Anti-Ubap2 was used for ELISA (USCN Life Science Technology; E94593Hu 96test). Anti-Ubap2 was used for ELISA (antibodies-online GmbH; ABIN6971034). Anti-OCN was used for ELISA (Merck; HBN1A-51K).<br>ALP-conjugated anti-DIG Fab antibody was used for zebrafish experiments (1:5000; Roche; 11093274910). Alexa fluorophore 488 secondary antibody was used for zebrafish experiments (1:500; Invitrogen; A11001).<br>Rabbit polyclonal PE anti-mouse/human CD11b antibody was used for flow cytometry (1:1000; BioLegend; 101207; Lot. B172191).<br>Mouse monoclonal FITC anti-CD90/Thy1 antibody was used for flow cytometry (1:1000; Abcam; ab226; GR3202875-8).

Validation	For the detection of Ubap2, 50 µg of mouse osteoblast lysate was analyzed using SDS-PAGE on 8% polyacrylamide gels. The proteins were electroblotted onto PVDF membranes for 1 h 30 m. The membrane blots were blocked with 2.5% BSA mixed with 2.5% skim milk for Ubap2 from Abcepta, and 5% BSA for Ubap2 antibodies from Abcam and Bethyl, and incubated with the primary antibodies in a cold room overnight. After washing with PBST, the membranes were incubated with the secondary antibodies for 1 h at room temperature .<br>[https://www.abcepta.com/products/BP12773a-UBAP2-Antibody-N-term-Blocking-peptide]<br>[https://www.abcam.com/products/primary-antibodies/ubap2-antibody-ab197083.html]<br>[https://www.thermofisher.com/antibody/product/UBAP2-Antibody-Polyclonal/A304-626A]<br>For the detection of E-cadherin, 20 µg of mouse osteoblast lysate was analyzed using SDS-PAGE on 8% polyacrylamide gels. The proteins were electroblotted onto PVDF membranes for 1 h 30 min. The membrane blots were blocked with 5% BSA and incubated with primary antibodies in a cold room overnight. After washing with PBST, the membranes were incubated with secondary antibodies for 1 h at room temperature.<br>[https://www.ptglab.com/products/E-cadherin-Antibody-20874-1-AP.htm]<br>For the detection of Fra1, 20 µg of mouse osteoblast lysate was analyzed using SDS-PAGE on 12% polyacrylamide gels. The proteins were electroblotted onto PVDF membranes for 1 h. The membrane blots were blocked with 5% BSA and incubated with primary antibodies in a cold room overnight. After washing with PBST, the membranes were incubated with secondary antibodies for 1 h at room temperature.<br>[https://www.scbt.com/p/fra-1-antibody-c-12]<br>Ubap2, E-cadherin1, Fra1, and β-actin proteins were visualized at approximately 110–120 , 110, 35, and 43 kDa, respectively .<br>Based on high identity levels of human and zebrafish ubap2 proteins (>50%), the polyclonal ubap2a antibody used (Abcepta; AP12773a; 1:10) is expected to specifically cross-react with zebrafish Ubap2 proteins. Consistent with this expectation, no immunostaining was observed without the primary UBAP2 antibody (Supplementary Fig. 10), and the intensity of Ubap2 fluorescence staining with the UBAP2 antibody decreased upon ubap2a knockdown (Supplementary Fig. 11).

Eukaryotic cell lines

Policy information about cell lines and Sex and Gender in Research

Cell line source(s)	Mouse pre-osteoblast MC3T3-E1 cells were purchased from RIKEN Cell Bank in Japan.<br>Monocytes were harvested from bone marrow cells of the femur of 6 -week-old BALB/c mice.<br>HEK293TN cells were purchased from System Biosciences (LV900A-1).<br>HeLa cells were purchased from the Korean Cell Line Bank.
Authentication	The increase in osteoblastogenesis and expression of osteoblast markers (Alp, Sp7, and Bglap) by inducing osteoblastic differentiation in MC3T3-E1 cells demonstrates that MC3T3-E1 cells are a pre-osteoblastic cell line. Primary-cultured monocytes were validated using a immunophenotypic analysis with the CD11b antibody (BioLegend, San Diego, CA, USA) using the FACS Aria III cell sorter (BD Biosciences) and FACS Diva software (BD Biosciences). 293TN cell line was transformed with the SV40 large T antigen for the high titer production of lentiviral particles and featured the neomycin resistance marker for stable propagation.
Mycoplasma contamination	Mycoplasma contamination was tested in all cell lines used in this study using the BioMycoX Mycoplasma PCR detection kit (Cell Safe; D-100; Lot. MD010620), and the results were negative.
Commonly misidentified lines<br>(See ICLAC register)	Not used.

Animals and other research organisms

Policy information about studies involving animals; ARRIVE guidelines recommended for reporting animal research, and Sex and Gender in Research

Laboratory animals	The animals were maintained on a regular dark–light cycle of 12:12 h at 22°C–24°C with 40%–60% humidity and free access for food and water throughout the experimental period. To isolate primary monocytes, 6-week-old female BALB/c mice (n = 9) were used. To prepare primary-cultured monocytes, the 6-week-old mice were euthanized using CO2 gas. The femoral bones of mice were collected and the bone marrow was flushed into α-MEM supplemented with 100 ng/mL receptor activator of nuclear factors κB ligand (RANKL; PEPROTECH; #315-11) and 10 ng/mL macrophage colony-stimulating factor (M-CSF; PEPROTECH; #315-02). Zebrafish AB strain at 1–6 days post-fertilization were used; sex was not determined during these stages.
Wild animals	This study did not involve wild animals.
Reporting on sex	For the primary-cultured mouse cell experiment, sex was determined based on phenotypic assessments. Only female mice were used under the same conditions as human research.
Field-collected samples	This study did not involve samples collected from the field.
Ethics oversight	Primary-cultured mouse cell experiments were approved by the Institutional Animal Care and Use Committee (IACUC) of the Ajou University School of Medicine (approval number: IACUC No. 2014-0066) and conducted in accordance with the institutional guidelines established by the Committee. Zebrafish husbandry and animal care were performed in accordance with the guidelines from the Korea Research Institute of Bioscience and Biotechnology (KRIBB) and approved by KRIBB-IACUC (approval number: KRIBB-AEC-21117).

Note that full information on the approval of the study protocol must also be provided in the manuscript.

Flow Cytometry

Plots

Confirm that:

☒ The axis labels state the marker and fluorochrome used (e.g. CD4-FITC).

☒ The axis scales are clearly visible. Include numbers along axes only for bottom left plot of group (a 'group' is an analysis of identical markers).

☒ All plots are contour plots with outliers or pseudocolor plots.

☒ A numerical value for number of cells or percentage (with statistics) is provided.

Methodology

Sample preparation	This study did not involve samples collected from the field.
Instrument	Primary-cultured monocytes were validated using the FACS Aria III cell sorter (BD Biosciences).
Software	FACS Diva version 8.0.3 software (BD Biosciences) was used for analysis.
Cell population abundance	FITC fluorescence was negatively detected in CD90-conjugated monocyte samples. It indicates the absence of contamination of mesenchymal stem cells.
Gating strategy	Untreated monocyte (CD11b- and CD90-) samples were analyzed for setting the FSC/SSC gates of cell population.

☒ Tick this box to confirm that a figure exemplifying the gating strategy is provided in the Supplementary Information.
